# Activation of transcription factor circuity in 2i-induced ground state pluripotency is independent of repressive global epigenetic landscapes

**DOI:** 10.1093/nar/gkaa529

**Published:** 2020-06-25

**Authors:** Ruchi Shukla, Heidi K Mjoseng, John P Thomson, Simon Kling, Duncan Sproul, Donncha S Dunican, Bernard Ramsahoye, Tuempong Wongtawan, Fridolin Treindl, Markus F Templin, Ian R Adams, Sari Pennings, Richard R Meehan

**Affiliations:** MRC Human Genetics Unit, Institute of Genetics and Molecular Medicine, WGH, University of Edinburgh, Edinburgh EH4 2XU, UK; Newcastle University Centre for Cancer, Biosciences Institute, Newcastle University, Newcastle-upon-Tyne NE2 4HH, UK; MRC Human Genetics Unit, Institute of Genetics and Molecular Medicine, WGH, University of Edinburgh, Edinburgh EH4 2XU, UK; MRC Human Genetics Unit, Institute of Genetics and Molecular Medicine, WGH, University of Edinburgh, Edinburgh EH4 2XU, UK; NMI Natural and Medical Sciences Institute, Tübingen University, Reutlingen, Germany; MRC Human Genetics Unit, Institute of Genetics and Molecular Medicine, WGH, University of Edinburgh, Edinburgh EH4 2XU, UK; MRC Human Genetics Unit, Institute of Genetics and Molecular Medicine, WGH, University of Edinburgh, Edinburgh EH4 2XU, UK; Centre for Genomic and Experimental Medicine, Institute of Genetics and Molecular Medicine, WGH, University of Edinburgh, Edinburgh EH4 2XU, UK; Centre for Cardiovascular Science, Queen's Medical Research Institute, University of Edinburgh, Edinburgh EH16 4TJ, UK; NMI Natural and Medical Sciences Institute, Tübingen University, Reutlingen, Germany; Pharmaceutical Biotechnology, Tübingen University, Tübingen, Germany; NMI Natural and Medical Sciences Institute, Tübingen University, Reutlingen, Germany; Pharmaceutical Biotechnology, Tübingen University, Tübingen, Germany; MRC Human Genetics Unit, Institute of Genetics and Molecular Medicine, WGH, University of Edinburgh, Edinburgh EH4 2XU, UK; Centre for Cardiovascular Science, Queen's Medical Research Institute, University of Edinburgh, Edinburgh EH16 4TJ, UK; MRC Human Genetics Unit, Institute of Genetics and Molecular Medicine, WGH, University of Edinburgh, Edinburgh EH4 2XU, UK

## Abstract

Mouse embryonic stem cells (mESCs) cultured with MEK/ERK and GSK3β (2i) inhibitors transition to ground state pluripotency. Gene expression changes, redistribution of histone H3K27me3 profiles and global DNA hypomethylation are hallmarks of 2i exposure, but it is unclear whether epigenetic alterations are required to achieve and maintain ground state or occur as an outcome of 2i signal induced changes. Here we show that ESCs with three epitypes, WT, constitutively methylated, or hypomethylated, all undergo comparable morphological, protein expression and transcriptome changes independently of global alterations of DNA methylation levels or changes in H3K27me3 profiles. *Dazl* and *Fkbp6* expression are induced by 2i in all three epitypes, despite exhibiting hypermethylated promoters in constitutively methylated ESCs. We identify a number of activated gene promoters that undergo 2i dependent loss of H3K27me3 in all three epitypes, however genetic and pharmaceutical inhibition experiments show that H3K27me3 is not required for their silencing in non-2i conditions. By separating and defining their contributions, our data suggest that repressive epigenetic systems play minor roles in mESC self-renewal and naïve ground state establishment by core sets of dominant pluripotency associated transcription factor networks, which operate independently from these epigenetic processes.

## INTRODUCTION

Mouse embryonic stem cells (mESCs) can be derived from the inner cell mass (ICM) of blastocysts following their *in vitro* culture (1,2). Culture medium developed for mESCs contains leukemia inhibitory factor (LIF) in the presence of serum or bone morphogenetic protein 4, which induces phosphorylation and activation of downstream transcription factors Stat3 and Smad1 (3,4). Cultured mESCs have considerable self-renewing capacity via expression of pluripotency genes that maintain their identity; they can contribute to germline chimeras and can differentiate into all three primary germ layers *in vitro* (5). Pluripotency in mESCs depends upon the coordinated action of a gene regulatory network assembled from transcription factors (e.g. Oct4, Sox2, Nanog: the OSN network), which is subject to modulation by multiple signalling pathways in response to environmental cues that support self-renewal or initiate differentiation (6,7). Under serum/LIF (serum) culturing conditions, a fine balance between renewal and pro-differentiation signals results in populations of mESCs that are heterogeneous and metastable, exhibiting a dynamic equilibrium in expression states for several pluripotency factors (8). Importantly, during the process of derivation of mESCs from pre-implantation stage embryos the cells adopt mature heterochromatin and DNA/histone modifications that deviate from their *in vivo* ICM counterparts (9). Overall, the cellular and chromatin identity of mESCs in serum cultures partly resembles that of ICM cells, epiblast cells and germ cells (10,11).

Culturing mESCS in 2i/LIF conditions containing inhibitors of MEK/ERK and GSK3β leads to a more homogeneous population, which more closely resemble ICM cells in terms of gene expression and epigenetic signatures, termed the naïve ground state (11). Transfer of mESCs from serum to 2i conditions consolidates developmental naïvety by endowing these cells with additional pluripotency features (12). These include enhanced *Nanog* and *Klf2* expression and epigenetic changes, such as global DNA hypomethylation and redistribution of H3K27me3 (a repressive histone modification mark) from bivalent CpG island promoters (13). It has been proposed that 2i conversion of mESCs represents a remodelling of the epigenome in concert with a reconfiguration of the gene regulatory network, which enables unbiased developmental plasticity (14).

Although DNA methylation is essential for the maturation of an embryo, it is striking that globally hypomethylated mESCs lacking de novo and maintenance cytosine methyltransferases cultured in serum grow robustly and self-renew (15–17). TKO (*Dnmt3a^−^^/^^−^*, *Dnmt3b^−^^/^^−^*; *Dnmt1^−^^/^*^−^) cells express typical markers of pluripotency (*Oct4*, *Rex1*, *Fgf4* and *Nanog*) to the same extent as their wild-type (WT) J1 counterparts, strongly suggesting that DNA methylation is dispensable for mESC maintenance. Lack of DNA methylation restricts the capacity of these cells to differentiate into early embryonic stages but is permissive for extraembryonic lineages *in vivo* (18). Transcriptional repression associated with DNA methylation is essential for maintaining somatic cell fates and identity (18,19). In serum cultures, different pluripotent states defined by high-low expression states of *Stella*, *Nanog* and *Rex1*, respectively, are correlated with the relative global methylation status of mESCs. This suggests a potential role for DNA methylation in the generation and maintenance of variability in stem cell populations (8,13,20). However, the direct contribution of DNA hypomethylation in driving mESCs to a naïve gene expression pattern is still unclear. In addition, the subsequent global redistribution of repressive H3K27me3 marks observed in hypomethylated 2i mESCs may be causally required to stabilize and maintain the ground state, or it may be an indirect consequence of decreased DNA methylation levels (13).

To explore the potential contributions of DNA methylation in relation to mESC identity and stability in serum and 2i cultures, we compared the response of WT mESCs with either constitutively hypomethylated or constitutively methylated mESCs. We observed a similar response in cell morphology, attainment of uniform expression of pluripotency genes (*Nanog* and *Esrrb*) and deep silencing of differentiation genes. We identified a set of core transcriptional changes that occur in the transition to 2i in all three epitypes, suggesting that these changes affect signalling regulated genes and occur independently of DNA methylation state in mESCs. In addition, we identified a subset of CpG island genes that undergo signal-induced transcriptional changes that coincide with a depletion of H3K27me3 in all three cell types under 2i conditions, however, H3K27me3 is not required for their silencing in serum cultured mESCs. Our data suggest that altered patterns of DNA methylation and H3K27me3 do not define naive state identity, which is primarily dictated by transcriptional and signalling networks. Instead these epigenetic transitions may be part of dynamic chromatin state changes prior to differentiation, when epigenetic regulatory mechanisms have a role in defining gene expression states (21). This general conclusion is in broad agreement with recent work that studied the impact of PRC2 function in the transition of mESCs to a 2i ground state, in which loss of Eed (a PRC2 component) to prevent H3K27me3 deposition had a minimal effect on the 2i transcriptome, implying that it is largely dispensable for establishment of the ground state (22,23). This chimes with our recent work showing that although DNA methylation has a role in shaping major aspects of PRC directed 3D genome organization in mESCs, this also does not contribute to maintenance of the 2i ground state (24).

## MATERIALS AND METHODS

### Cell culture

All cell lines used were generated from male WT J1 mESCs that were originally derived from the 129S4/SvJae strain, except *Dnmt1^tet/tet^* cells, derived from R1 mESCs. mESCs were cultured in serum conditions as previously described (24,25), except ESGRO LIF (Millipore) was used at 500 U/ml for all lines apart from *Dnmt1^tet/tet^*mESCs which were cultured at 1000 U/ml. WT J1 mESCs were grown on Mitomycin C inactivated SNLP feeder cells in serum cultures, all other cell lines were grown feeder free on 0.2% gelatin-coated flasks. 2i culturing conditions were as previously described (24,25); inhibitors were used at 1 μM PD0325901 (MEK inhibitor, Stemgent) and 3 μM CHIR99021 (GSK3 inhibitor, Stemgent) and ESGRO LIF was used at 1000 U/ml. Doxycycline-inducible Prdm14 overexpressing TKO cells were established by transfecting PB-TET-FlagPrdm14-IRES-Neo, PB-CArtTA Adv, pCAG-Pbase and pGG131 (pCAG-DsRed-IRES-Hygro) (26) into TKO cells using Fugene 6 HD (Promega) followed by sorting red cells using a BD FACSAria flow cytometer; single cell clones were expanded and characterised. KOVI-3l and 3B3l mESCs were generated as described in McLaughlin *et al.*^24^. *Dnmt1^tet/tet^* doxycycline regulateable mESCs were a gift from R. Chaillet lab, for derivation procedure see Borowczyk *et al.* (27). *Dnmt1^tet/tet^* doxycycline regulateable mESCs were treated with 10μM EPZ6438 (EZH2 inhibitor) for 9 days.

### siRNA knockdown

RNAiMAX (ThermoFisher) was used to transfect 2 × 10^5 mESCs/6 well and 1 × 10^5^ mESCs per 12-well (on coverslips coated with 0.2% gelatin) with 50 nM siRNA Tet1 plus 50 nM siRNA Tet2 or 100 nM scrambled siRNA (see Supplementary information, Table S1 for sequences). mESCs were cultured post transfection in either serum for 72 or 48 h in serum followed by 24 h in 2i; medium was changed every day.

### Embryoid body differentiation

mESCs were detached with trypsin and spun down prior to resuspension in mESC basal medium with 20% FCS and plating as 200 cells/20 μl as hanging drop cultures. After 2 days cells aggregated and EBs were cultured in suspension in mESC basal medium for 3 days prior to 3–6 days of adherent culture on 0.2% gelatin on glass coverslips. Patches of cardiomyocytes were recorded, and EBs were fixed with 4% PFA for 15 min and stored at 4°C covered in PBS prior to staining.

### Differentiation to epiblast stem cells

6 × 10^4^ mESCs/six-well were seeded in standard serum conditions on human fibronectin (Millipore, FC010) for 24 h and subsequently cultured in EpiSC culture medium supplemented with N2 (Gibco, 17502048) and B27 (Gibco, 17504044) consisting of a 1:1 mixture of DMEM F-12 (Thermo Fisher, 21331020) and Neurobasal medium (Thermo Fisher, 21103049), 0.1 mM non-essential amino acids (SIGMA), 2 mM l-glutamine, 0.1 mM beta mercaptoethanol (Thermo Fisher), Activin A (Peprotech, 120-14E) at 20 ng/ml and FGF2 (R&D systems, 3139-FB) at 10 ng/ml. Cells were passaged every 3–5 days with accutase (Gibco, A1110501) and reseeded as clusters of cells at 4 × 10^4^ cells/six-well, which resulted in a stable Epiblast stem cell line after several passages.

### RT-qPCR

Total RNA was extracted using an RNeasy kit (Qiagen), following manufacturer's protocol and using Qiashredder columns (Qiagen) to homogenize the samples and RNase free DNase kit (Qiagen) to perform DNA digestion. 1 μg RNA was converted to cDNA using random primers (Promega) and Superscript III (Thermo Fisher) following manufacturer's protocol. qPCR reactions with gene specific primers (see Supplementary Table S1) were carried out using SYBR green (Roche or Thermo Fisher) on a Lightcycler 480 System II (Roche). TBP was used for normalisation; relative quantification was done by the 2^-dCt^ method. Three biological replicates were used for statistics and standard deviation was calculated to generate error bars following MIQE guidelines (Supplementary Table S3).

### Western blotting

Protein was extracted using RIPA buffer (Thermo Fisher) supplemented with protease inhibitor cocktail (Roche) followed by boiling in 1× LDS sample buffer and 1× reducing reagent (both Thermo Fisher) for 5 min at 95°C. 30, 15 or 7.5 μg of protein was loaded per sample and run in 4–12% Bis–Tris gels (Thermo Fisher) in 1× MOPS or MES buffer (Thermo Fisher). Dry blotting was performed using an iBlot™ device (Thermo Fisher) and PVDF iBlot™ gel transfer stacks. Membranes were blocked in 0.1% Tween-20 in PBS with 1:10 dilution of western blocking reagent (Thermo Fisher) for a minimum of 45 min at room temperature followed by primary antibody incubation at 4°C overnight and secondary antibody incubation for 1 h at room temperature (for antibodies and dilutions see; supplementary information, Table S2). HRP stained western blots were developed using SuperSignal West Pico Chemiluminescent substrate (Thermo Scientific) and imaged using an ImageQuant LAS 4000 (GE Healthcare). Near infrared stained blots were imaged on an Odyssey Fc system (LI-COR, Nebraska, USA).

### Immunocytochemistry

mESCs on glass coverslips coated with gelatin were stained using standard immunocytochemistry protocols. Briefly, mESCs were fixed with 4% PFA, 10 min and incubated overnight with primary antibodies (see; supplementary information, Table S2) at 4°C, followed by incubation with appropriate Alexafluor-conjugated secondary antibodies (Thermo Fisher) at room temperature for 1 h. Nuclei were labelled using DAPI (4′,6-diamidino-2-phenylindole). Imaging was done using a Zeiss Axioscope 2 microscope with Zeiss optics or a Nikon A1 Confocal system with Zeiss optics. Embryos were stained following procedure previously described (9). Briefly, blastocyst stage embryos were fixed in 4% PFA overnight at 4°C followed by permeabilization in 0.2% Triton X-100 (Sigma-Aldrich) in PBS for 30 min and two washes with PBS-0.01% Tween (Sigma-Aldrich; PBST) prior to blocking with 5% donkey serum (Sigma-Aldrich) in PBST for 2 h at room temperature. Primary antibodies (see; supplementary information, Table S2) were incubated overnight at 4°C in 1% donkey serum in PBS, followed by three washes in PBST. Secondary antibodies conjugated to either FITC or TRITC (Jackson Laboratories) were used at 1:200 for 1 h at room temperature, followed by washes. Samples were counterstained with DAPI in Vectashield mounting medium (Vectashield). Images were captured with a Zeiss laser confocal microscope (LSM510 Meta) and LSM software.

### Alkaline phosphatase staining

Alkaline phosphatase staining was performed using Alkaline phosphatase staining kit II (Stemgent) according to manufacturer's instructions and imaged using a Nikon Eclipse Ti-S microscope.

### Methylation analysis

Genomic DNA was extracted using DNeasy Blood and Tissue kit (Qiagen) following manufacturer's protocol or standard phenol chloroform precipitation and eluted in water. DNA was treated with RNase A/T1 Cocktail (Ambion) overnight at 37°C followed by ethanol precipitation. Quantitation of 5mC in genomic DNA was done by isocratic high performance reverse phase liquid chromatography (HPLC) as previously described (28) with the following alterations. A Dionex UM 3000 HPLC system was used complete with a column chiller, C18 column (250 mm × 4.6 mm 5 μM APEX ODS, Grace Discovery Sciences), and column guard (Phomenex). The mobile phase was 50 mM ammomium phosphate (monobasic) pH4.1. The column was chilled to 8°C to improve peak separation. Deoxyribonucleotides (dNMPs) were detected at their extinction maxima using a Dionex 3000 multiple wavelength detector: dCMP, 276 nm; 5mdCMP, 282 nm. Quantifications were calculated from the area under each peak using the respective extinction coefficients (dCMP, 8.86 × 10^3^; 5mdCMP 9.0 × 10^3^).

### Bisulfite sequencing

Bisulfite sequencing was carried out as described (29). For details of primers utilised see; Supplementary information, Table S1. Bisulfite conversion of DNA was performed using the EZ-DNA methylation gold kit (ZymoResearch), products were cloned into pGEM-T Easy (Promega) and minimum 24 clones were picked per condition for plasmid preparation followed by sequencing using BigDye, version 3.1 chemistry (Thermo Fisher). Bisulfite sequencing DNA methylation analysis (BISMA) was used to calculate percent methylation in bisulfite sequencing reads.

### Agilent expression array and data analysis

Total RNA was isolated using RNeasy kit (Qiagen) and Cy3 labelled aRNA was prepared using an Amino Allyl MessageAmp II aRNA kit (Ambion). Samples were hybridised to SurePrint G3 mouse GE 8 × 60k microarrays (Agilent) and scanned using NimbleGen MS200 (Roche). Results were analysed with custom-written scripts implemented in R (http://www.R-project.org). Differential expression was calculated using the Bioconductor package linear models for microarray data (limma v3.20.8). *P*-values were corrected for multiple testing using the Benjamini–Hochberg test and probes with *P* < 0.05 were deemed significant.

### meDIP

meDIP was carried out as described (30). In short, 20 μg of genomic DNA was diluted in TE to 400 ul and sonicated using a Covaris plus sonicator to 150–700 bp range with a mean of 300 bp. 6 μg of fragmented DNA was diluted to 450 μl in TE and denatured by incubation in a thermoshaker for 10 min at 99°C. 10% of the sample was taken at this stage as an input fraction and stored at 4°C. Samples were incubated with 15 μl of 5mC antibody (Eurogentec) overnight at 4°C. M280 Dynabeads were used for immunoprecipitation. DNA was purified using a PCR clean up kit (Qiagen) and samples were eluted into 20 μl; 10 μl was subjected to whole genome amplification using a SEQXE WGA kit (SIGMA) per manufacturer's instructions, except no SYBR green was added at the amplification step and samples were amplified for a total of 18 cycles at which point the volume required to get 2.1 μg of the lowest sample was the volume used from each sample for further primer removal and clean up using a PCR clean up kit (Qiagen). We performed proton sequencing on libraries made from 100 ng of DNA per sample, using the Ion XpressPlus Fragment Library Kit (Thermo Fisher). DNA was end repaired, purified, ligated to Ion-compatible barcoded adapters (Ion Xpress™ Barcode Adapters 1–96, Thermo Fisher), followed by nick-repair to complete the linkage between adapters and DNA inserts. The adapter-ligated library was then amplified (10 cycles) and size-selected using two rounds of AMPure XP bead (Beckman Coulter) capture to obtain fragments of ∼100–250 bp. Samples were then pooled at a 1:1 ratio and sequenced on an Ion Proton P1 microwell chip (Thermo Fisher).

### RNA sequencing

Total RNA was extracted from mESCs using an RNeasy kit (Qiagen). RNA was analysed using a bioanalyser and confirmed to be above RIN 9. Samples were treated with DNase (Ambion), and sample integrity verified on the Agilent Bioanalyser with the RNA Nano-chip. Illumina Tru-seq paired end strand specific sequencing (Illumina, USA) was carried out on a NextSeq-550 sequencer (Edinburgh Wellcome trust Clinical Research Facility, Western General Hospital, Edinburgh, UK). 500 ng of Total RNA underwent ribosomal RNA depletion prior to purification (EPZ6438 treated *Dnmt1*^tet/tet^ mESCs) or PolyA-selection (J1, TKO and 3B3l mESCs), fragmentation, random hexamer cDNA generation and purification with AMPure XP beads (Beckman-Coulter, USA). Multiple indexing adapters were ligated to ds cDNA with subsequent hybridisation onto flow cells, and DNA fragment enrichment by 15 cycle PCR for sequencing. Completed libraries were quantified by qPCR using the KAPA Illumina Library Quantification Kit (Illumina, USA) before multiplexing in two equimolar pools and running on two flow cells on the Illumina NextSeq 550. Resulting FastQ files were mapped to the reference genome (mm10) using the Tophat alignment tool (V2) on Illumina Basespace software and reads per kilobase per million (RPKM) scores calculated for each gene. Differential gene expression was carried out using DEseq with cut offs of log_2_ fold change >2 and adjusted *P*-values <0.05 within replicates applied.

### H3K27me3 ChIP-seq

ChIP was performed exactly as described (24). Sequencing libraries and Ion proton sequencing was carried out as described for MeDIP-seq above. For details of antibodies see; Supplementary information, Table S2.

### High content western blotting – DigiWest

DigiWest was performed essentially as described in Treindl *et al.* (31). Briefly; cell pellets were lysed in RIPA Buffer containing protease Inhibitor Mix M (Serva), PhosSTOP (Roche Applied Science) and PMSF (Thermo Scientific), incubated for 30 min on ice and protein was quantified using a Pierce BCA Protein Assay Kit (Thermo Fisher Scientific). Samples were stored at −80°C until further use. The NuPAGE SDS-PAGE gel system (Thermo Fisher) was used for protein separation and blotting; 8 μl protein per sample was separated using 4–12% Bis–Tris gels and transferred onto PVDF membranes (Millipore). For high content western analysis, the DigiWest procedure and data analysis was performed as described in Treindl *et al.* (31). In brief; data generated by a Luminex instrument were analysed using a dedicated analysis tool. To compare different samples, obtained values were normalized to β-actin; log_2_ transformed and analysed in MEV 4.9.0 software. Two factor ANOVA was performed in which factor 1 was defined as 2i treatment and factor two as cell type (J1 versus TKO versus 3B3l). *P*-value based on 1000 permutations was set to 0.005 and hierarchical clustering based on Euclidean Distance was performed. Significant results were plotted in a heatmap. DigiWest data are composed of 96 individual measurements derived from 96 molecular weight fractions per sample and all values obtained for a given antibody were used to create images that mimic a Western blot image. Background subtracted Luminex data were scaled to values from 0 and 1 where the highest measured signal intensity was set to 1. Scaled data were loaded into the MEV 4.9.0 software package, and a graph was generated by applying a greyscale colour scheme; 0 (white) to 1 (black). Heatmaps were saved as images and transferred to Photoshop wherein a Gaussian diffusion was applied with a radius of half the element height. DigiWest Luminexreads can be found in; supplementary information, Dataset 2.

### Bioinformatic processing and analysis of meDIP-Seq and H3K27me3 ChIP-seq datasets

#### Mapping and data normalization

Analysis was done as described previously (30). In short, reads were mapped to reference genome using Torrent TMAP software. Data was binned into 200 bp windows across the genome and normalized first by total read count and then sequencing noise removed by subtracting the matched input sequence (sheared non-immunoprecipitated DNA).

#### Peak finding and mapping

Windows of enrichment were defined as any 200 bp window where the signal was enriched over background input noise. Peaks of 5mC and H3K27me3 were called based on threshold levels from J1-serum cells. We defined peaks as regions where at least 2 windows (each 200 bp) in a three-window region (600 bp) were above the 95th percentile of 5mC scores from the J1-serum dataset. These peaks were mapped to one of six unique genomic compartments (promoter core: TSS ±100 bp, promoter proximal: TSS + 1 kb, promoter distal: TSS + 1 kb to + 2 kb, exonic, intronic or inter-genic: not associated with any of the above) as defined from Refgene annotations.

#### Sliding window analysis of 5mC states over regions of interest

Average patterns of DNA modifications and histone tail marks were plotted across a series of genomic features (promoters ±2 kb, gene bodies ±25% total gene length, Oct4/Sox2/Nanog binding sites ±100% element length) using a sliding window based approach. This calculates average levels of each modification across a certain step size relative to coordinates of choice and average patterns across these features are then plotted. Oct4/Sox2/Nanog binding sites were taken as defined in Galonska *et al.* (22).

### Retrotransposon analysis

J1, TKO and 3B3l mESC RNA-seq was processed as described (24). For retrotransposon analysis, adaptors were removed from RNA-seq reads using TrimGalore! 0.4.1 (paired end, illumina, stringency 3), and aligned to the mm9 mouse genome using TopHat 2.1.0 (very sensitive, inner distance 23 ± 56, no coverage search, max multihits 1). Read co-ordinates were intersected with UCSC genome browser RepeatMasker track co-ordinates using BEDTools 2.25.0, filtered to ensure that each pair or singleton was assigned to only one repeat location, and the number of sequences belonging to each type of repeat summed. Repeats belonging to LTR, LINE and SINE Repeatmasker classes that were significantly upregulated (FDR< 0.05, logFC > 0) were identified using the edgeR package in R. Read counts between samples were normalised relative to the number of reads mapping to the genome.

## RESULTS

### mESCs reset to a naïve ground state in 2i independently of their initial epigenetic state

**Figure 1.**
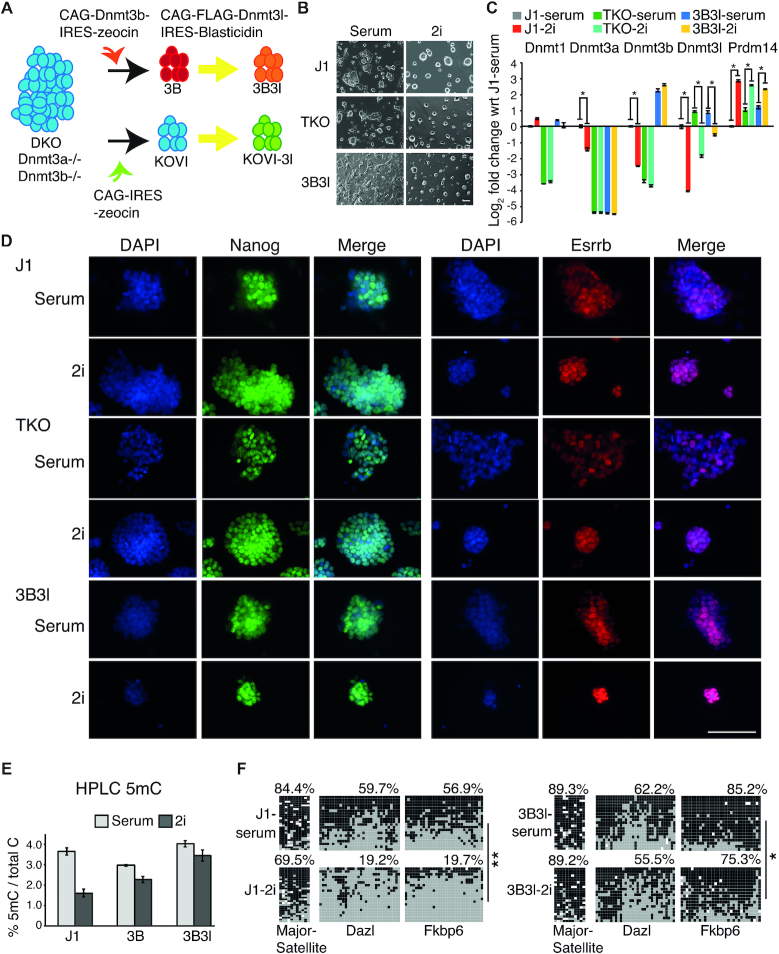
J1, TKO and 3B3l mESCs achieve ground state in 2i. (**A**) Schematic showing cell line derivation. An expression construct for the co-factor *Dnmt3l* was added to *Dnmt3a/3b* knockout mESCs rescued by exogenous constitutive expression of *Dnmt3b* (3B) to create a *Dnmt3a/3b* knockout expressing both *Dnmt3l* and *Dnmt3b* (3B3l). *Dnmt3l* was also added to a to *Dnmt3a/3b* knockout mESCs (KOVI) to create KOVI-3l. (**B**) Morphology images of mESCs in indicated culture conditions. Scale bar 100 μm. (**C**) Expression levels of indicated Dnmt's and Prdm14. For each gene, fold change with respect to (wrt) average J1-serum probe value is represented (as log2) with SEMs. * indicates *P* < 0.05 (unpaired *t*-test). (**D**) Immunocytochemistry images of indicated conditions showing Nanog (green) and Esrrb (red) with DNA counterstaining (DAPI, blue) and merge. Scale bar 100 μm. (**E**) HPLC quantification of 5mC levels of indicated mESCs, bars represent mean ± SD of 2–3 biological replicates. (**F**) Bisulfite sequencing data for indicated gene promoters and cell lines, black: methylated CpG, grey: un-methylated CpG, white: missing data. Numbers represent percentage of methylated CpGs. * (black: methylated CpG, grey: un-methylated CpG, white: missing data). *P < 0.05, ** *P* = 0.00578, two2-tailed Mann–Whitney *U*-test.

To investigate the role of repressive epigenetic marks in ground state pluripotency we cultured WT, hypomethylated, and constitutively methylated mESCs and analysed their response to 2i conditions. As a hypomethylated cell type we utilised TKO mESCs lacking functional *Dnmt3a*, *Dnmt3b* and *Dnmt1* (15). To generate constitutively methylated 2i/mESCs, we utilised *Dnmt3a/3b* knockout mESCs rescued by exogenous constitutive expression of *Dnmt3b* and *Dnmt3l*, as detailed in the cell line derivation schematic, hereafter termed 3B3l (Figure 1A) (24,32). WT J1, TKO and 3B3l mESCs were cultured in 2i medium for 14 days. All three cell types showed characteristic mESC morphologies in serum conditions and acquired archetypal three-dimensional dome-shaped colony morphology after 3 days of 2i exposure (Figure 1B) (25). Expression of a key marker of 2i transition, *Prdm14*, was upregulated in all three cell lines (Figure 1C) (33). In WT JI mESCs Transcripts corresponding to *de novo* methyltransferases (*Dnmt3a*, *Dnmt3b* and *Dnmt3l*) were downregulated while the maintenance methyltransferase (*Dnmt1*) remained unchanged, as observed by microarray analysis and confirmed by RT-qPCR (Figure 1C and Supplementary Figure S1a). As expected, only the *Dnmt3l* transcript was downregulated in TKO cells, as the *Dnmt3a*, *Dnmt3b* and *Dnmt1* genes are inactivated. In addition, we confirmed retention of *Dnmt3b* and *Dnmt3l* transcripts in 3B3l mESCs (Figure 1C and Supplementary Figure S1b). We also detected homogeneous expression of Nanog and Esrrb in WT J1, TKO and 3B3l mESCs cultured in 2i by immunocytochemistry, in contrast to heterogeneous staining in their serum cultured counterparts (Figure 1D).

To analyse changes in DNA methylation in WT J1, TKO and 3B3l mESCs when converted to a 2i mediated ground state, we performed high performance liquid chromatography (HPLC) quantification of total 5mC levels, which revealed various degrees of hypomethylation in 2i (Figure 1E). As reported previously, 5mC levels in 2i are reduced by 50% in WT mESCs (J1–2i) (Figure 1E). Notably, 3B3l cells cultured in 2i (3B3l-2i) exhibited global DNA methylation levels equivalent to WT J1-serum cells (Figure 1E). Hence, maintenance of *Dnmt3b* and *Dnmt3l* expression can prevent cells from exhibiting global hypomethylation despite increased *Prdm14* levels during 2i adaptation (Figure 1C and Supplementary Figure S1b). Loss of DNA methylation upon 2i exposure is linked with Prdm14 directed repression of *Dnmt3a*/*Dnmt3b*/*Dnmt3l* or alternatively, a deficiency of UHRF1 and H3K9me2, required for maintenance methylation (34–36). Although we observed the reported 2i induced reduction of UHRF1 and H3K9me2 levels in WT mESCs that is hypothesised to reduce Dnmt1 activity, these alterations were not replicated in TKO-2i or 3B3l-2i mESCs (Supplementary information, Figure S1c, d) (34). The observation that overexpression of *Dnmt3b* and *Dnmt3l* (or deletion of *Prdm14*, see below) can maintain methylation levels in 2i mESCs is incompatible with the primary role proposed for UHRF1 in regulating global DNA methylation levels in 2i mESCs (34,35). Bisulfite sequencing analysis of loci (major satellite, *Dazl* and *Fkbp6* promoters) known to undergo hypomethylation in 2i demonstrated that 3B3l-2i methylation was maintained at most of these sites (major satellite, *Dazl*) or underwent a slight decrease (*Fkbp6* 10% decrease, *P* < 0.05, two-tailed Mann–Whitney *U*-test), but still remained significantly more highly methylated than in J1–2i cells (*Fkbp6* 75.3% in 3B3l-2i compared with 19.7% in J1–2i, *P* < 0.00001, two-tailed Mann–Whitney *U*-test) (Figure 1F).

To further characterize WT J1, TKO and 3B3l mESCs cultured in 2i we performed several experiments to confirm their pluripotency and 2i mediated signalling responses. All three epitypes exhibit heterogeneous alkaline phosphatase activity in serum and this increases to 100% homogeneous alkaline phosphatase activity in mESCs cultured in 2i (Supplementary information, Figure S1e). To analyse changes in signalling pathways following 2i culture we utilised the western blotting analysis system DigiWest to simultaneously assay 72 proteins (31). This confirmed the absence of Dnmt1 protein in TKO mESCs and maintenance of Dnmt3l in 3B3l-2i at levels significantly above J1-2i and TKO-2i (Supplementary information, Figure S1f). As expected, culturing mESCs in 2i resulted in upregulation of β-catenin, one of the downstream targets of the GSK3β pathway and downregulation of phospho-c-Myc, a downstream target of the MEK pathway (Supplementary information, Figure S1f) (25). Hence, relevant signalling pathways are intact in all three cell lines utilised in the study. Several other proteins exhibit similar expression changes in WT J1, TKO and 3B3l mESCs based on culture conditions e.g. vimentin, which may account for changes in cell shape during 2i adaptation (Supplementary information, Figure S1f). Moreover, ANOVA analysis of significant analytes (*P* < 0.005) resulted in clustering of DigiWest samples according to culture conditions (Supplementary information, Figure S1g, Dataset 2); implying that signal transduction pathways that respond to 2i in WT J1, TKO and 3B3l mESCs induce similar protein expression and modification perturbations. In addition, we found that J1 and 3B3l mESCS were capable of forming differentiated embryoid body (EB) outgrowths when plated onto gelatin. TKO mESCs formed EBs but failed to form significant outgrowths and very few cells stained positive for α-smooth muscle actin (mesodermal marker) and Tuj1 (ectoderm) (Supplementary information Figure S2a and data not shown). This is in agreement with previous observations that lack of DNA methylation in TKO mESCs leads to impaired differentiation to ecto- and mesoderm (18). WT J1 and 3B3l mESCs formed ectoderm and mesoderm lineages; both generated beating cardiomyocytes staining positive for α-smooth muscle actin, as well as cells positive for Tuj1 (Supplementary information, Figure S2b). It is note-worthy that the α−smooth muscle actin marker not only reports on a mesodermal lineage but also attests the earliest differentiation from pluripotent mouse ESCs into mesendoderm, (ME) the intermediate stage equivalent to the embryonic primitive streak, from which both mesoderm and endoderm are derived (37). However, we did not observe significant endodermal marker expression for either cell type with this differentiation protocol; perhaps indicating that a more directed differentiation approach may be required to generate this lineage with these cell lines (data not shown). In addition, WT J1 and 3B3l mESCs both generated a stable epiblast stem cell line when treated with appropriate cytokines, however TKO mESCs were unable to differentiate to this lineage (Supplementary information, Figure S2c). Taken together this data suggests that 3B3l mESCs can transition to a naive ground state and differentiate into specialised cell types unlike TKO cells, which are differentiation impaired.

**Figure 2.**
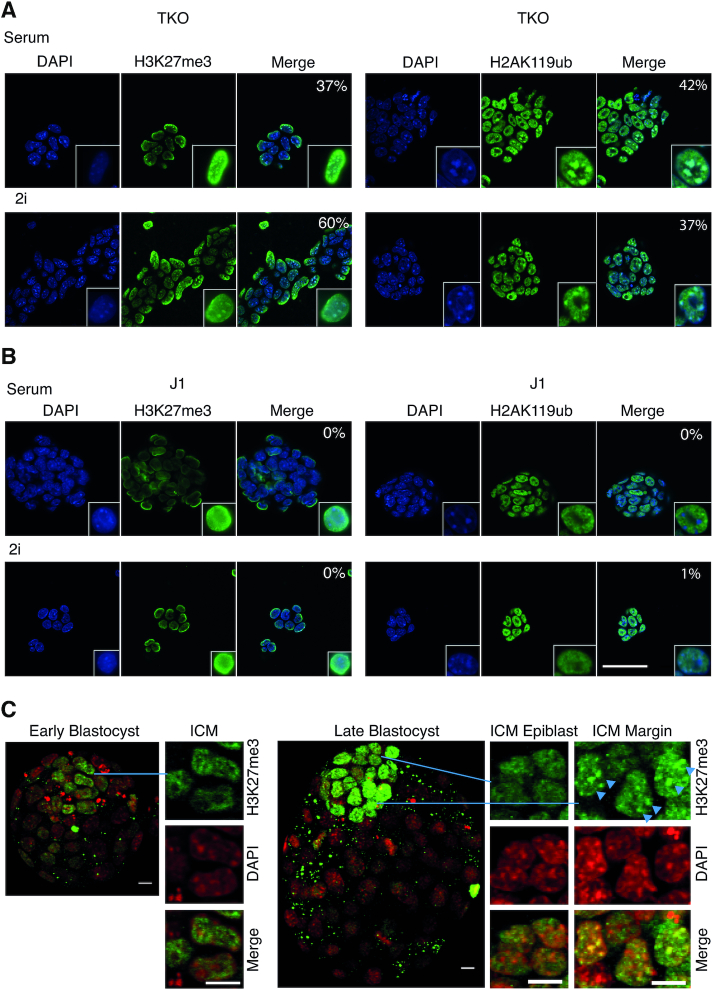
Epigenetic state of heterochromatin in mESCs and H3K27me3 dynamics during mouse blastocyst development. (**A**, **B**) ICC of H3K27me3 (green) and H2AK119Ubiquitin (green) in indicated cells with DAPI (blue) and merge (turquoise represents overlapping signal). White outlined nuclei are ‘zoomed in’ images of representative nuclei. Numbers represent the percentage of cells where green foci overlap with DAPI stained heterochromatin. Scale bar 50 μm. (**C**) ICC for H3K27me3 (green) with DAPI (pseudocoloured red). Whole and part embryo confocal projections and highlighted cells from the inner ICM and the ICM margin (yellow represents overlapping signal), as indicated; scale bars 10 μm. Arrowheads indicate three cells showing heterochromatic H3K27me3 staining.

In order to investigate alterations in repressive chromatin marks in response to DNA methylation changes and 2i signalling we performed immunocytochemistry (ICC) for H3K27me3 and H2AK119ub in WT J1, TKO and 3B3l mESCs cultured in serum and 2i, as well as in early and late blastocysts. As reported previously, both H3K27me3 and H2AK119ub are redirected to pericentric heterochromatin (PCH) in a high proportion of TKO cells under serum conditions (Figure 2A) (38). A similar proportion exhibited PCH localisation for both modifications in TKO-2i cells (Figure 2A). In contrast, no significant PCH association of H3K27me3 and H2AK119ub was observed in J1-2i or 3B3l-2i cells (Figure 2B; Supplementary information, Figure S3). This contrasts with a recent report suggesting that 2i-ESCs are distinguished from other pluripotent cells by a prominent enrichment in H3K27me3 and low levels of DNA methylation at PCH (39). However we note that Tosolini and colleagues culture their 2i-mESCs on Laminin which also leads to the appearence of H3K27me3 in conjunction with H3K9me3 at PCH in serum grown mESCs, whereas we follow the original SOP of Ying *et al.* and grow ESCs on gelatin coated plastic (25). It is possible that the ability of Lamins to confer proliferative stimulation on mESCs may cause additional changes that affect H3K27me3 deposition when mESCs are transitioned to 2i (40). To verify the situation *in vivo*, we isolated blastocyst stage embryos and stained them for H3K27me3 (9). Early and late male blastocysts show a marked increase in H3K27me3 levels in the ICM, in contrast to very low trophectoderm (TE) levels (Figure 2C). Early ICM patterns display euchromatic distribution away from DNA-dense foci similar to WT J1 mESCs. This pattern is maintained in the epiblast; however, in some cells of the late ICM margin proximal to the blastocoele, H3K27me3 foci colocalise with regions of high DNA density in a pattern similar to TKO cells (Figure 2C). This indicates redistribution of H3K27me3 might be a response to developmental cues that involves DNA hypomethylation of the ICM (41,42). During the meiotic prophase of spermatogenesis, H3K27me3 is also observed to accumulate on PCH from the mid pachytene stage onward, during which DNA methylation reprogramming occurs as evidenced by hyomethylated minor satellite repeats (43,44).

### Inhibition of MEK1/2 and GSK3β is necessary to drive TKO cells to the naïve state

As a reduction in DNA methylation has been postulated to be a hallmark of the naïve state, we analysed TKO mESCs to address whether they would convert more easily to the ground state than WT mESCs. We checked the sensitivity of J1 and TKO cells to the two inhibitors at various dilutions in defined media (SFES) with a constant LIF concentration. Both cell lines responded comparably and neither achieved ground state morphology when cultured in diluted 2i medium (Supplementary information, Figure S4a). We observed comparable trends in key transcript changes in both J1 and TKO mESCs in diluted 2i, but alterations were not to the same extent as in complete 2i (Supplementary information, Figure S4b). We also evaluated the effect of individual inhibitors, PD0325901 and CHIR99021. TKO cells acquired a 3D-dome shaped colony morphology similar to 2i adaptation, upon exposure to CHIR99021 + LIF (CH), while exhibiting very distinct needle-shaped flat cell morphology upon PD0325901 + LIF (PD) treatment (Supplementary information, Figure S4c, d). *Nanog* expression was lower in TKO cells incubated with CH alone compared to PD or 2i cultured cells (Supplementary information, Figure S4e). Prolonged culture in no inhibitor medium (SFES) or single inhibitor (CH or PD) resulted in decreased cell viability and spontaneous differentiation compared to 2i cells, in agreement with reports for WT cells (Supplementary information, Figure S4d) (25). Associated gene expression changes (notably *Dnmt3l*, *Eomes* and *Lin28a*) did not occur to the same extent with single inhibitors as compared to inhibiting both pathways (Supplementary information, Figure S4f).

As *Prdm14* is suggested to be a key player in driving cells to the 2i ground state, we generated a TET-inducible *Prdm14* TKO mESC line to test if its overexpression in hypomethylated mESCs is sufficient to drive these mESCs into a ground state (Supplementary information, Figure S5a, b) (35). Upon induction of Prdm14 no morphological changes were observed in TET-ON-Prdm14 TKO clones (Supplementary information, Figure S5c). Additionally, although direct targets of *Prdm14* such as *Dnmt3l* and *Lefty2* were down regulated, we did not observe transcript changes associated with 2i adaptation such as upregulation of *Dmgdh* (highest expression change in 2i versus serum in wild-type mESCs) or *Eomes* (stem cell maintenance gene) and downregulation of *Pax6* and *Fgf15* (differentiation genes), in any of the TET-ON-Prdm14 TKO clones irrespective of the level of *Prdm14* overexpression (Supplementary information, Figure S5d) (45). This suggests *Prdm14* upregulation and 5mC depletion alone is not sufficient to reprogram serum cultured mESCs to a ground state in terms of morphology (Supplementary information, Figure S5c) or gene expression (Supplementary information, Figure S5d).

### Gene expression patterns are determined by culture conditions

**Figure 3.**
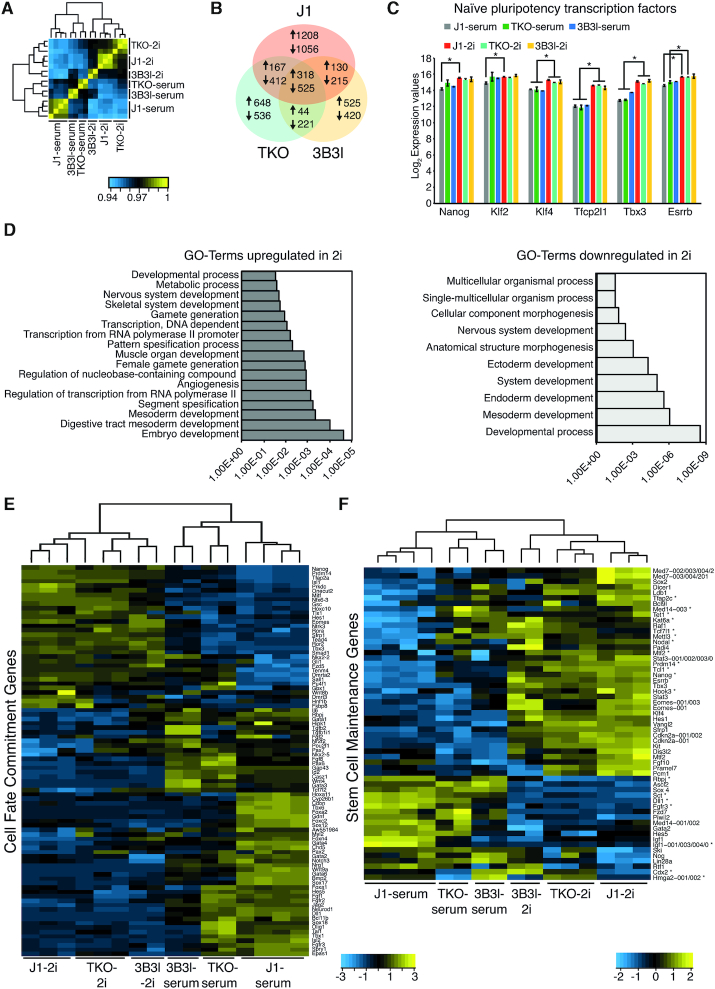
Gene expression changes in mESCs upon culturing in 2i. (**A**) Heatmap showing Pearson correlation and clustering of microarray data for indicated cell lines and culture conditions. (**B**) Venn diagram showing the number of genes changing in expression (FC ≥ 2, *P*.adj. ≤ 0.05 eBayes (limma), Benjamini–Hochberg corrected) in J1-2i (red), TKO-2i (green) and 3B3l-2i (yellow) compared to their serum counterparts. (**C**) Log_2_ expression values of indicated genes in mESCs in serum and 2i. Bars represent mean ± SD. * indicates *P* < 0.05 (unpaired *t*-test). (**D**) GO-Term analysis showing genes up- or down-regulated in J1, TKO and 3B3l mESCs. (**E**), Heatmap representing expression levels of ‘Cell Fate Commitment’ genes differentially expressed (FC ≥ 1.5, p.adj. ≤ 0.05 eBayes (limma), Benjamini-Hochberg corrected) in J1-2i compared to J1-serum in indicated cells. * marks genes which are differentially expressed (FC ≥ 2, *P*.adj. ≤ 0.05 eBayes (limma), Benjamini–Hochberg corrected) between TKO-serum and J1-serum. (**F**), Same as E for ‘Stem Cell Maintenance’ genes.

In order to delineate gene expression changes caused by 2i signalling from gene expression changes caused by a difference in DNA methylation, we analysed gene expression in WT J1, TKO and 3B3l mESCs cultured in serum and 2i. Pearson correlation analysis of expression profiles revealed robust clustering based on culture conditions rather than genotype (Figure 3A). The number of differentially expressed genes between TKO and WT J1 mESCs in 2i compared to serum culture conditions is reduced and consolidates as 3.3 times less upregulated and 2.9 times less downregulated genes (Supplementary information, Figure S6a, b). Moreover, in spite of being hypomethylated in 2i, the transcriptome of WT J1-2i mESCs differs greatly from that of TKO-serum (Supplementary information, Figure S6c). In agreement with previous reports, we observed a large number of genes (>3500) differentially expressed in 2i wild-type cells compared to serum conditions (1823 up- and 2208 down-regulated, fold change ≥2, *P*.adj. ≤ 0.05, eBayes (limma), Benjamini–Hochberg corrected) (Figure 3B; Supplementary information, Dataset 1) (13,35,46). There is an overlap of 843 genes between all three 2i cell lines that exhibited similar changes in expression (318 up- and 525 down-regulated) (Figure 3b; Supplementary information, Dataset 1). Moreover, transcription factors associated with naïve pluripotency are upregulated in WT J1, TKO and 3B3l in 2i compared to respective serum cells; with the exception of *Klf2* which exhibited increased expression in TKO and 3B3l mESCs in serum (Figure 3C). Importantly, these factors are equivalently expressed in all three epitypes in 2i. Functional pathway analysis of the common gene list upregulated in 2i revealed enrichment of genes related to germ cell and embryonic development, while genes downregulated in 2i are linked with differentiation (Figure 3D). Based on pathway analysis, we focused on genes associated with the GO terms ‘Cell Fate Commitment’ (CFC: GO:0045165) and ‘Stem Cell Maintenance’ (SCM: GO:0019827), which were differentially expressed (FC ≥ 1.5, *P*.adj < 0.05, eBayes (limma), Benjamini–Hochberg corrected) between WT J1-serum and J1-2i mESCs. These gene sets showed a similar trend in TKO and 3B3l mESCs following adaptation to 2i (Figure 3E, F; Supplementary information, Figures S6d and S7) and we also observed this in WT and *Prdm14^−^^/^^−^* mESCs, (Supplementary information, Figure S6e, f) (35). In addition, hierarchical clustering analysis linked these cell types together based on culture condition rather than genotype (Supplementary information, Figure S6g). Our results emphasize that mESCs can achieve a ‘transcriptional ground state’ in 2i through enhanced expression of a number of pluripotency associated genes, including *Nanog, Esrrb and Klf4*, irrespective of global methylation status, and that hypomethylation is dispensable for this transition (Figure 3F).

### Epigenetic changes in the 2i mediated ground state

**Figure 4.**
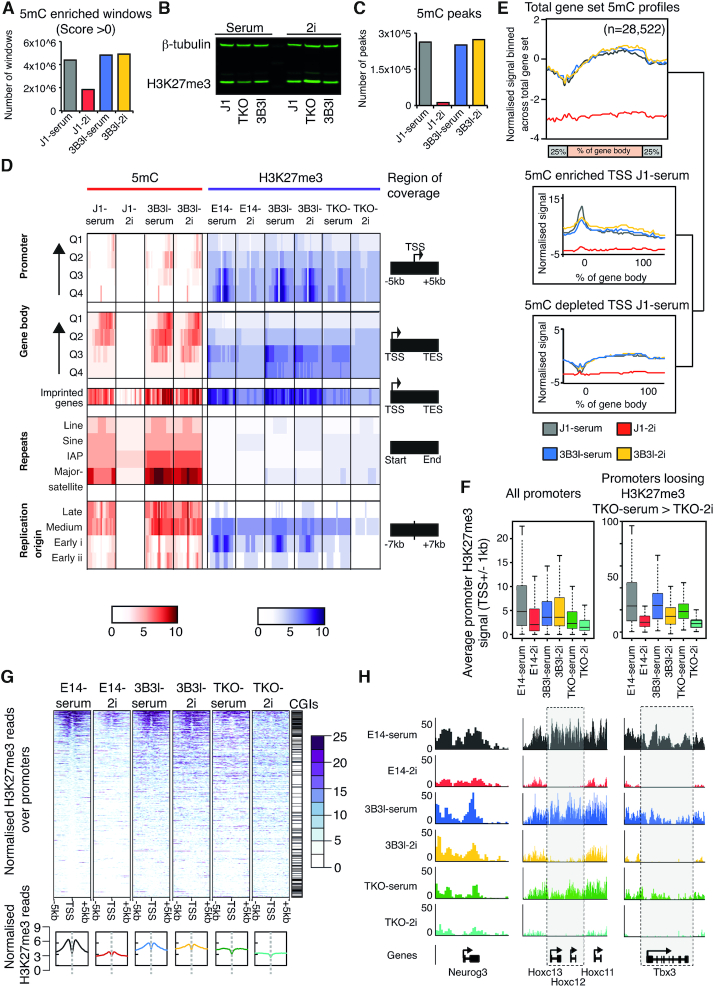
5mC and H3K27me3 modifications in J1, 3B3l and TKO mESCs in serum and 2i. (**A**) Windows enriched in 5mC relative to input sequence for indicated cell lines and conditions. (**B**) Fluorescent western blot of whole cell protein extracts showing levels of H3K27me3 for indicated cell lines cultured in serum or 2i. β-tubulin was used as a loading control. (**C**) Total number of 5mC peaks (see materials and methods) for indicated cell lines and conditions. (**D**) Heatmap indicating 5mC (red) and H3K27me3 (purple) levels; 0–10, see scale, for indicated cell lines and conditions across indicated genomic features, covering regions as described. Q4-Q1 indicates low to high gene expression. (**E**) Sliding window analysis of average genic 5mC patterns across indicated cell lines. Plots represent length normalised gene bodies with flanking regions (±25% of total gene length). Stratification of genic patterns by promoter 5mC enrichment (upper box) or depletion (lower box). Numbers of genes per set are shown above. (**F**) Boxplots representing average H3K27me3 signal on promoters (±1 kb from TSS) for all promoters or promoters preferentially losing H3K27me3 in TKO-2i relative to TKO-serum. * indicates *P* < 0.05 (Wilcoxon test). (**G**) Heatmap representing normalised H3K27me3 reads across promoters (±5 kb TSS). Bottom graphs represent averaged profiles. Heatmaps are ranked by E14 serum levels. (**H**) Examples of H3K27me3 pattern across *Neurog3*, *Hoxc13, 12, 11* and *Tbx3* for indicated cell lines and culture conditions.

To study functional consequences of DNA methylation rearrangement in the reprogramming of mESCs to ground state, we generated genome-wide patterns of 5mC in WT J1 and 3B3l mESCs cultured in serum and 2i by genome wide MeDIP-seq assays (30). We validated the levels of 5mC enrichment across the genome in J1 and 3B3l epitypes and report upon an ∼50% reduction in the number of both 5mC enriched loci (termed windows, see material and methods) and HPLC quantified 5mC scores in J1-2i mESCs (Figures 1E and 4A). The number of enriched windows in 3B3l-serum was similar to those in WT J1-serum; as expected this cell line did not exhibit the typical loss of 5mC upon 2i exposure (Figure 4A). Interestingly, although global levels of 5mC differ between the epitypes upon transition to ground state, by western blot there was no obvious change in total H3K27me3 levels in WT J1 and 3B3l mESCs in serum or 2i in agreement with two reports, but H3K27me3 levels were higher in TKO-2i cells (Figure 4B) (13,39). In contrast, a recent proteomic study found higher H3K27me3 levels in WT mESCs cultured in 2i conditions; despite this they also report, by ChIP-seq, a redistribution of H3K27me3 profiles in 2i culture conditions (23). Although we report an overall reduction in 5mC levels within these cells (as evident from a drop of 5mC peaks to 4.7% in the J1-21 cells with respect to the serum state. Figure 4C), the underlying genomic patterns remain largely unchanged (Figure 4D; Supplementary information, Figure S8a, b)

To refine our assessment, we interrogated 5mC and H3K27me3 patterns across a number of functional genomic compartments (Figure 4). Focusing first on promoter and gene body regions, we discovered that although levels and patterns of 5mC are lost upon transition to 2i in WT J1 cells, these are maintained across the same sets of promoters and gene bodies in J1-serum, 3B3l-serum and 3B3l-2i mESCs (Figure 4A and C–E). Analysis of locus specific patterns also revealed that 5mC marked promoters identified in WT J1 serum mESCs remain similarly marked in both 3B3l-serum and 2i cells (Figure 4D, E). To analyse H3K27me3 patterns we utilized a published E14 mESC dataset as our WT reference data (GSE23943) as their deposition at bivalent promoters is reported to be dependent on global DNA methylation levels (13,42,47). H3K27me3 patterns are typically reduced upon transition to 2i in WT E14 mESCs but broadly maintained over promoter and genic regions in the constitutively methylated cell line in 2i (Figure 4D, F). To delineate 5mC mediated changes from 2i signalling changes in H3K27me3 distribution; we compared patterns of H3K27me3 between TKO-serum and TKO-2i mESCs. Interestingly, we note a reduction in the H3K27me3 signal, which manifests in loss from a number of promoter elements (Figure 4F, G). Stratification of the total promoter set based on regions which lose H3K27me3 between TKO-serum and TKO-2i revealed a subset of 2i specific, DNA methylation independent regulation loci across all epitypes (Figure 4F). Visualization of these loci (specific examples; *Neurog3*, *Hoxc12–13* and *Tbx3*) uncovered a strong loss of H3K27me3 levels in 2i conditions irrespective of global DNA methylation state—highlighting a small set of common 2i signalling induced epigenetic changes in all three mESC epitypes (Figure 4H).

Analysis over enhancer and pluripotency factor binding elements revealed that such loci are typically depleted in 5mC and H3K27me3 in both serum and 2i conditions for all three epitypes (Figure 4D; Supplementary information, Figure S8b) (22). Analysis over annotated replication origin sites revealed a general loss of 5mC in WT J1-2i conditions but higher levels of 5mC at medium and late replicating sites in both WT J1-serum, 3B3l-serum and 3B3l-2i mESCs (Figure 4D) (48). Similarly, there is a general loss of H3K27me3 in WT E14-2i cells with higher levels of H3K27me3 present in early replication origins in WT E14-serum, 3B3l-serum and 3B3l-2i lines (Figure 4D). Additionally, DNA methylation is lost at repeat sequences (including major satellite and LINE-1 retrotransposons) in WT J1-2i but maintained in 3B3l-2i mESCs (Figure 4D).

**Figure 5.**
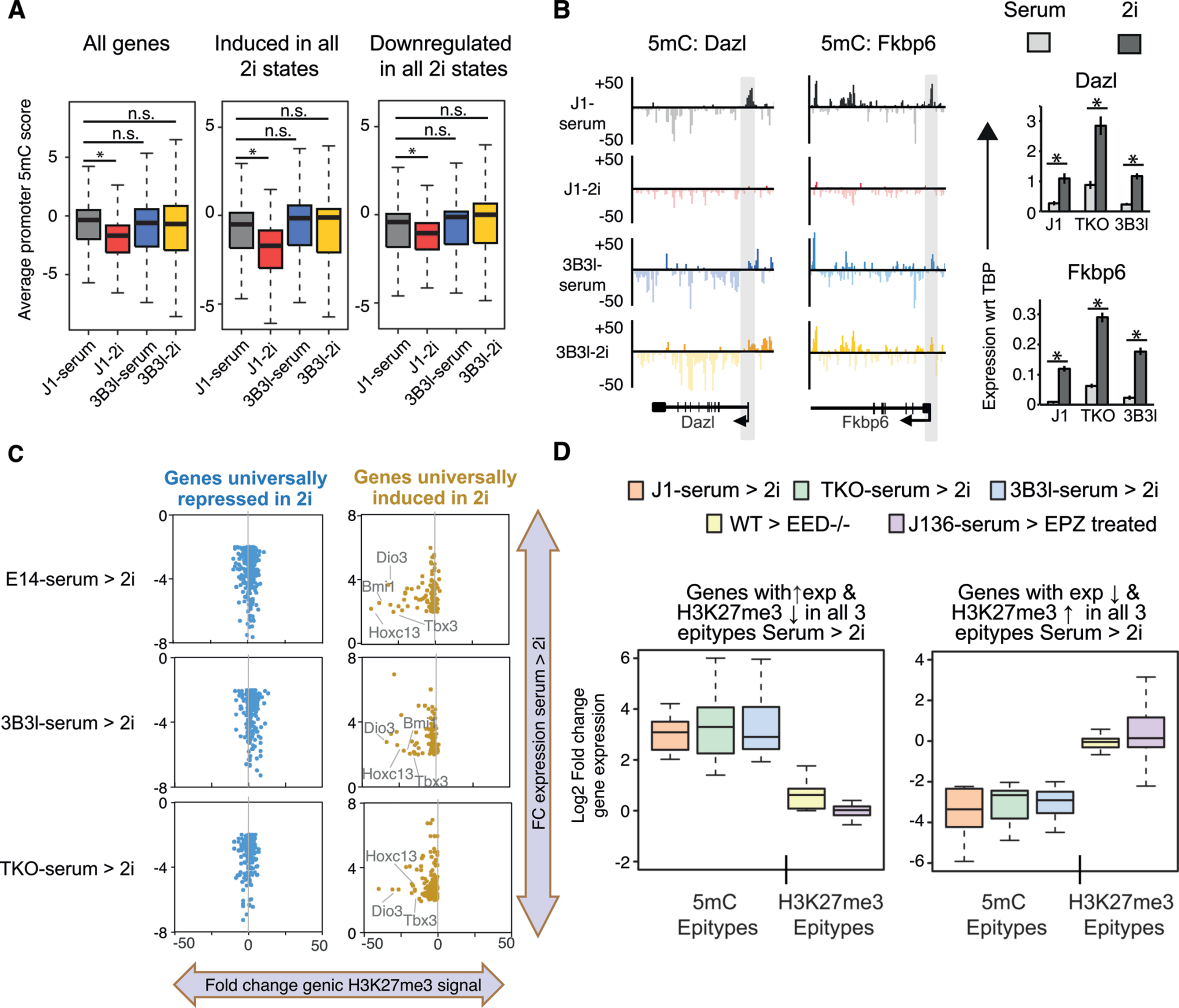
Epigenetic changes over transcriptionally altered genes in mESCs in serum and 2i. (**A**) Boxplots of average promoter 5mC in indicated cell lines over the total gene set, genes expressed under 2i conditions and genes repressed under 2i conditions. * indicates *P* < 0.05 (Wilcoxon test). (**B**) Examples of 5mC data at the promoters of the methylation regulated genes *Dazl* and *Fkbp6* alongside RT-qPCR data for serum and 2i conditions. Promoter regions are marked by grey shadows. Barcharts show expression analysis of indicated genes by RT-qPCR, values represent mean ± SD of expression with respect to (wrt) TBP. * indicates *P* < 0.05 (unpaired *t*-test). (**C**) Scatter plot of fold change in expression 2i-serum (y-axis) versus fold change in average genic H3K27me3 levels (2i-serum) in WT (left), 3B3l (centre) and TKO (right) cells. Yellow = 2i induced genes, blue = 2i repressed genes. (**D**) Boxplot detailing expression changes serum to 2i (log2 fold changes) across WT, TKO, 3B3l, *Eed^−^^/^^−^* and EPZ6438 treated *Dnmt1^tet/tet^*mESCs. Left: genes with overlapping H3K27me3 loss/expression gain in J1/TKO/3B3l mESCs. Right: genes with overlapping H3K27me3 gain/expression loss in J1/TKO/3B3l mESCs.

Analysis of RNA-seq data for WT J1, TKO and 3B3l mESCs cultured in serum and 2i revealed that some differences exist between culture conditions and epitypes tested in terms of retrotransposon expression (Supplementary information, Figure S8c–f). Consistent with previous observations, retrotransposon derepression in response to 2i or changes in DNA methylation was relatively modest in mESCs (Supplementary information, Figure S8c), and smaller in magnitude than observed for differentially expressed genes (49–51). A subset of 44 (28%) of the 156 retrotransposons upregulated when WT J1 mESCs transition from serum to 2i is also upregulated when TKO and 3B3l mESCs transition from serum to 2i (Supplementary information, Figure S8d). These retrotransposons, which include RLTR13B2::ETNERV3-int and RLTR44C::RLTR44-int long terminal repeats and associated internal sequences, are upregulated in 2i regardless of methylation state (Supplementary information, Figure S8f). Some elements in this subset, such as RLTR10D::IAP-d-int, appear to exhibit a combinatorial response to 2i and DNA methylation such that they are upregulated in response to hypomethylation and further upregulated in response to 2i (Supplementary information, Figure S8f). These elements exhibit similar behaviour to *Dazl* and *Fkbp6* genes (Figure 5B), although it is also possible that the mixed response of these elements reflects distinct responses of individual genomic copies of that repeat. Interestingly, most (149 of 203) of the retrotransposons that are upregulated in response to DNA hypomethylation in serum-cultured mESCs, including RLTR44B and MMERVK10D3_LTR, are not upregulated when WT J1 mESCs transition from serum to 2i (Supplementary information, Figure S8e, f). Thus, different retrotransposons respond to the 2i environment, changes in DNA methylation, or a combination of both in mESCs. Finally, imprinted loci exhibit a greater level of both 5mC and H3K27me3 retention during 2i reprogramming in both WT J1 and 3B3l mESCs, arguing that maintenance of 5mC levels may be functionally required at such sites (Figure 4D).

### Analysis of epigenetic changes over transcriptionally altered genes

As we observed a consistent change in transcription in all three epitypes when converted to 2i mediated ground state, we set out to elucidate whether specific changes to 5mC and H3K27me3 may be fundamental drivers of these changes. For the total arrayed gene set for all three cell types as well as for gene sets exhibiting strong changes in gene expression when cultured in 2i versus serum, we did not detect a relationship between alterations in promoter 5mC levels and the expression status of linked genes (Figure 5A & Supplementary information, Figure S9a). To validate this result, we selected two candidate methylation regulated genes; *Dazl* and *Fkbp6*, and examined the effect of 2i signalling on their expression in WT J1, TKO and 3B3l mESCs by RT-qPCR (Figure 5B) (13,52). These two transcripts are highly upregulated in TKO compared to WT J1 mESCs, supporting a role for promoter demethylation in their upregulation. However, they are both further upregulated in 2i in TKO and 3B3l mESCs and we observed comparable changes in Dazl protein levels by DigiWest analysis (Figure 5b and Supplementary information, Figure S9b). The promoters of these genes exhibited demethylation in WT J1-2i but maintained DNA methylation in 3B3l-2i as evidenced by meDIP and locus-specific bisulfite sequencing (Figures 5B and 1F). Hence, at transcript level, regardless of whether their promoters were hypomethylated or hypermethylated, both *Dazl* and *Fkbp6* responded in the same way to 2i signalling, resembling what we observed for a subset of 2i mediated changes in DNA repeat expression (Figure 5b and Supplementary information, Figure S8c–f).

We did observe a relationship between loss of H3K27me3 from genic regions of genes transcriptionally induced in 2i in all three epitypes; a relationship which was not observed over transcriptionally repressed genes (Figure 5C). To test whether these genes are regulated by 2i mediated signalling, we assayed their expression levels in mESCs with altered H3K27me3 levels; mESCs exposed to an inhibitor of the polycomb protein, EZH2 methyltransferase (EPZ6438), or through analysis of published expression data from polycomb regulatory complex mutant mESCs (*Eed^−^^/^^−^*) (53,54). We found that 2i induced genes, which lost H3K27me3 in WT, 3B3l and TKO mESCs, were not induced in these H3K27me3 perturbed serum cultured cells (Figure 5D; Supplementary information Figure S10), indicating that changes in expression of these genes are also direct consequences of 2i signalling and not due to changes in H3K27me3 deposition.

### Relationship between gene regulatory network and epigenetic landscape

**Figure 6.**
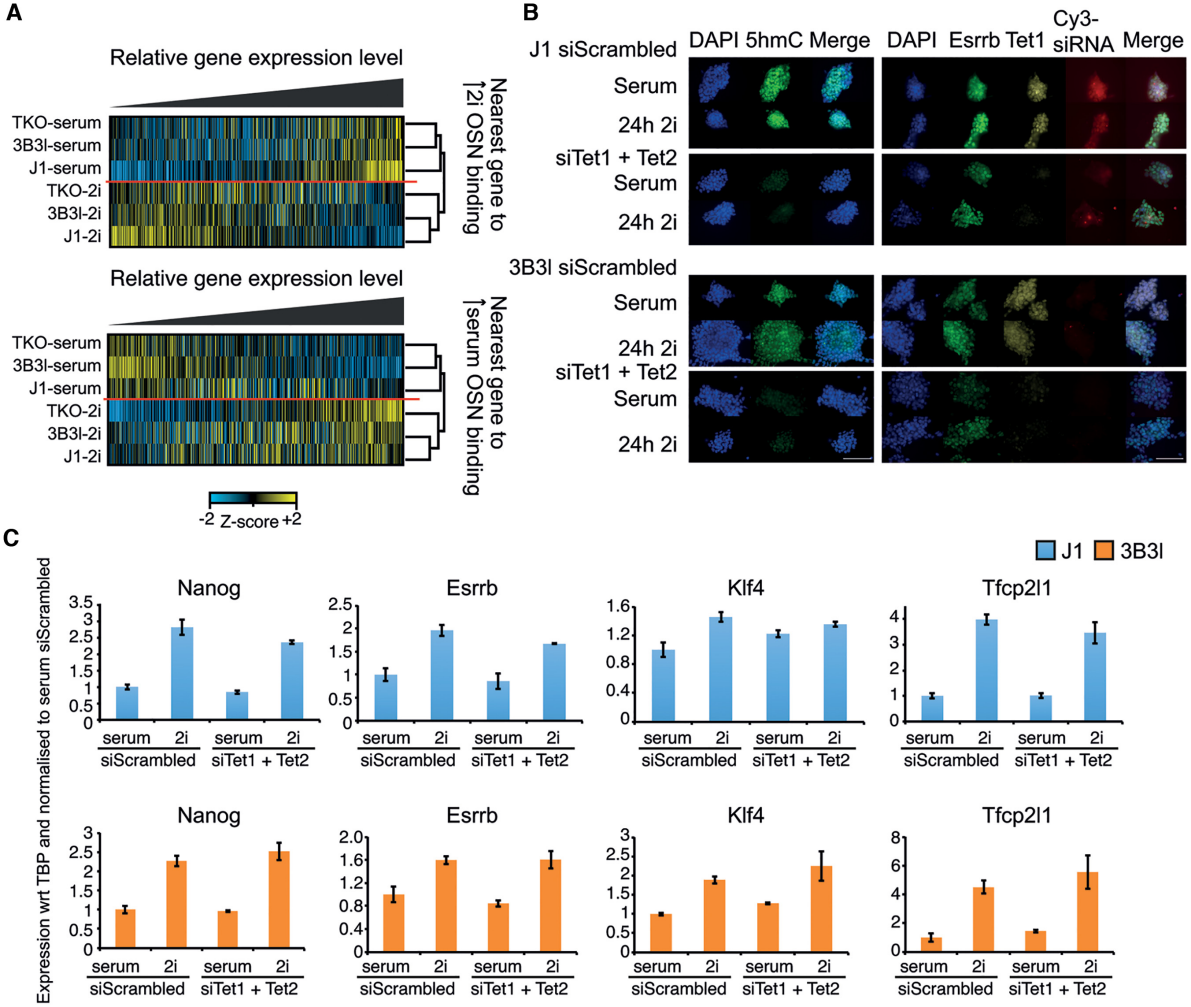
Relationship between, gene regulatory networks, epigenetic marks and culture conditions in mESCs. (**A**) *Z*-score normalised heatmaps ranked by relative expression level of the nearest gene to an OSN binding site (within ±10 kb) with increased binding in 2i (top) or increased binding in serum (bottom) for indicated cell lines. Hierarchical clustering groups by culture condition. (B, C) siRNA knockdown of Tet1 and Tet2 for indicated cell lines. (**B**) Immunocytochemistry for indicated cell lines. Scale bar is 100μm. Left: DAPI (blue), 5hmC (green) and merge. Right: DAPI (blue), Esrrb (green), Tet1 (yellow), Cy3-siRNA (red) and merge. (**C**) Expression analysis of indicated genes by RT-qPCR in J1–36 (blue) and 3B3l (orange) mESCs in 72 h serum or 48 h serum + 24 h 2i transfected with either scrambled siRNA or siRNAs for Tet1 + Tet2. Values represent mean ± S.D. of three technical replicates with respect to (wrt) to the housekeeping gene TBP (TATA Binding Protein), normalized to serum siScrambled values.

A recent study has shown that the differential binding of a core network of the pluripotency factors Oct4, Sox2 and Nanog (OSN) is important in reaching ground state following 2i exposure (22). However, it is not known how the epigenetic landscapes at these key regulatory sites are related to factor binding events and establishment of specific gene regulatory networks. We first set out to elucidate how the transcriptional landscape at genes near differentially bound OSN sites is affected during 2i transition by identifying the nearest neighbouring gene (first gene within 10 kb of an elevated or reduced OSN site in 2i, referred to as distal elements) and analysed their expression changes in our cell panel. We discovered a similar number of distal genes that are induced and repressed near 2i specific OSN sites upon 2i conversion but observed a large reduction in transcription at distal genes where OSN factors are reduced in 2i, arguing that transcription from a subset of genes is linked to binding of distal factors (Figure 6A and Supplementary information, Figure S11a). When we rank 2i driven changes in gene expression at these distal genes in WT J1 mESCs (i.e. log_2_ FC J1-serum > J1-2i, Supplementary Figure S11a), we observe similar transcriptomic changes following 2i conversion in WT J1, 3B3l and TKO mESCs (Figure 6A, Supplementary information, Figure S11a, b).

Enhancer elements and OSN binding sites are typically DNA hypomethylated, including in 3B3l mESCs. We therefore tested the hypothesis that active DNA demethylation could play a key role in the maintenance of this epigenetic state and that if perturbed would affect constitutively methylated mESCs to a greater degree than WT cells, potentially abrogating their ability to reach ground state. To do so we used an siRNA approach to reduce the levels of the DNA demethylation machinery members Tet1 and Tet2 in WT J1 and 3B3l mESCs cultured in serum or for 24 h in 2i (see Materials and Methods). Compared to scrambled siRNA controls, transfection of published siRNAs against Tet1 and Tet2 (Supplementary information, Figure S11c) was sufficient to reduce the 5hmC signal in these cells to undetectable levels by immunocytochemistry (Figure 6B) (55). This did not interfere with cell viability as evaluated by morphology, Esrrb staining or pluripotency marker expression assessed by qRT-PCR, indicating the 5hmC pathway was not required for the serum to 2i transition in WT J1 and 3B3l mESCs (Figure 6B, C).

## DISCUSSION

In this paper, we address some widely held assumptions regarding the epigenetic identity of ground state ES cells (56). Early observations reported that the transition of ES cells to ground state, dependent on ground state promoting signalling pathways, is accompanied by a reduction in DNA methylation levels (20,33). This major epigenetic state change, which also involves altered H3K27me3 deposition, was viewed as integral to the cells functional transition through gene expression changes (13). Surprisingly, however, this premise had not been fully tested. Our study reports three unexpected important new insights: the reduction in DNA methylation is not a prerequisite for ES cells to achieve the ground state transition; this global DNA hypomethylation does not lead to transcription alterations of the genes undergoing demethylation; and H3K27me3 redistribution induced by the reduction in DNA methylation does not lead to transcription changes of the genes affected, which are dependent on changes in transcriptional factor networks (22). Our conclusions obtained by a novel route of comparing serum to 2i transitions of wild type, hypomethylated and constitutively DNA methylated mESCs, are in alignment with work using PRC2 deficient mESCs that defined 2i induced transcription factor and proteomic changes respectively (22,23). These papers showed that PRC2 function is not required for mESC pluripotency, self renewal and the 2i transition. Significantly Van Mierlo *et al.* also observe in 2i cultured PRC2 deficient mESCs a relative increase in DNA methylation levels that appears to be due to enhanced protein expression of Dnmt3a, Dnmt3b and Dnmt3l (23). This observation again challenges the view that it is perturbation of the Dnmt1/Uhrf1 axis that is responsible for hypomethylation in 2i cultured mESCs (34). Consistent with a direct impact of PRC2 deficiency on DNA methylation levels in 2i cultured mESCs, hypermethylation is also observed at the CGI shores of a distinct class of bivalent genes in serum/LIF cultured PRC1/2 mutant mESCs (42). This implies that DNA methylation and Polycomb, two distinct epigenetic repression systems, have direct as well as indirect impacts on each other. Collectively our data and data from Van Mierlo *et al.* and Galonska *et al.* support the view that during the 2i transition changes in H3K27me3 and/or DNA methylation are secondary effects, which do not influence specific gene expression in 2i mESCs (22,23). These conclusions raise the question to what extent the epigenetic state of a cell defines its identity. In the case of embryonic stem cells the answer points to an epigenetic ground state that is decoupled from the pluripotency ground state, with both the gene regulatory network and the epigenetic network poised to interconnect at or after differentiation. Of relevance here is the previous suggestion that enhanced germline gene expression in ground state mESCs is primarily a consequence of global DNA hypomethylation (49). We tested this premise and found that patterns of gene expression including germline, are similarly dependent on 2i culture conditions, and occur independently of DNA methylation status. Our observations imply that global reduction of 5mC is not required for gene regulatory networks to orchestrate changes in transcription resulting in altered stem cell states. This is in line with the observation that in some species extensive reprogramming of DNA methylation in early embryogenesis is not observed (57,58).

### H3K27me3 regulated gene expression

All three mESC epitypes showed similar morphological changes, enhanced expression of pluripotency factors and signalling induced changes at a discrete set of H3K27me3 marked genes (e.g. *Tbx3*, *Hoxc12* and *Hoxc13*). Notably, expression of these H3K27me3 marked genes was not activated in serum in the absence of polycomb repression. This agrees with our recent work on the identification of DNA methylation mediated changes in chromatin compaction that occurs when WT mESCs are shifted from serum to 2i growth conditions (24). Chromatin decompaction at polycomb marked regions is prevented in 3B3l-2i mESCs when global DNA methylation is maintained, but inhibition of decompaction also does not appear to have phenotypic consequences. Similarly, polycomb dependent chromatin compaction in hypomethylated TKO cells is predicted to be already altered in serum conditions, but, as we have shown, does not significantly impact on the ability of these cells to transition to a ground state under 2i conditions. These observations support the idea that DNA methylation and polycomb processes are not instrumental for the transition of mESCs to a ground state (22,59). It is noteworthy that during early cleavage-stage mouse embryos, H3K27me3 is absent from promoters under hypomethylated conditions (42,60). It is only later that bivalent domains (H3K4me3/H3K27me3) and topological associated domains are newly established in the inner cell mass/trophectoderm, which constitutes the chromatin environment that supports the gene regulatory networks necessary for subsequent development (60,61). Our results widen the impact of the observation that H3K27me3 is dispensable for repression of bivalent genes and *de novo* silencing in 2i (22).

### DNA methylation regulated gene expression

Our data provide a set of core transcriptomic changes that occur independently of methylation in the conversion of mESCs to the ground state. Despite hypomethylated TKO-serum mESCs showing some intermediate gene expression states, they firmly cluster with WT-serum mESCs and have no less requirement for 2i signalling for conversion to a uniform ground state. Our orthologous experiments demonstrate that constitutive DNA methylation is also not an impediment to 2i adaptation. The impact of DNA methylation loss at promoter regions is relatively restricted here, but even at bona fide methylation dependent genes (*Dazl* and *Fkbp6*), their induction in 2i conditions occurs independently of whether their promoters are constitutively hypomethylated or methylated. The promoter methylation that can be directly overridden in mESCs contrasts with observations of its inhibitory effect in somatic cells to date, and needs further investigation of these different cellular contexts (52). Changes in retrotransposon expression in response to hypomethylation or 2i signalling are not dramatic in contrast to retrotransposon derepression in hypomethylated somatic cells, or mESCs defective in *Setdb1*-dependent histone methylation (29,50,51). Tellingly, neither *Prdm14* inactivation in WT cells, nor its overexpression in TKO mESCs affects 2i mediated transition to the naïve state (35). *Prdm14^−^^/^^−^* mESCs in 2i exhibit global levels of DNA methylation comparable to WT mESCs cultured in serum, which offers strong support that *Prdm14* is a key driver of DNA demethylation in 2i cultures, and not inhibition of Dnmt1 activity by a deficiency of UHRF1 and H3K9me2 factors (20,26,34). Analysis of changes in gene subsets related to stem cell maintenance and cell fate commitment in microarray data from *Prdm14*^−/−^ and WT mESCs in 2i with hierarchical clustering of these datasets are in complete agreement with the present study suggesting that the role of Prdm14 in antagonising FGFR signalling and repression of *de novo* Dnmt enzymes maybe incidental to ground state conversion (20). The characteristic changes that 3B3l mESCs undergo in 2i in terms of morphology, pluripotency marker homogeneity and gene expression changes in stem cell maintenance and cell fate commitment gene subsets supports this view. This indicates that serum/LIF associated patterns of DNA methylation and H3K27me3 are compatible with the 2i state. In addition, OSN enhancers associated with canonical Wnt and ERK signalling pathways appear to function equivalently in all three epitypes and in the case of 3B3l mESCs did not depend on Tet mediated demethylation for activity in 2i conditions. We conclude that reorganisation of core pluripotency factors in 2i is independent of the global epigenetic (DNA methylation and H3K27me3) state of the cells.

Our results raise new hypotheses regarding the role of DNA methylation and PRC2 (Polycomb Repressive Complex 2) in the 2i transition. In the blastocyst embryo, the early ICM state exists during a short-lived window in the lead-up to embryonic differentiation or the derivation of mESC lines, which are thought to acquire late epiblast characteristics in *in vitro* cultures (9). This is accompanied by higher levels of DNA methylation, more restricted *Prdm14* expression, fluctuating pluripotency gene expression and emergence of differentiation marks and heterochromatin maturation marks as well as a shorter cell cycle time (10,62). From this perspective, the conversion of serum mESCs to 2i conditions can be regarded as a reprogramming of these cells to an earlier pluripotent state, which does not maintain these late markers, but their presence is not a hindrance to regaining a 2i mediated ground state. This may be especially relevant to redefining the hallmarks of naïve pluripotency in other mammals that may have distinct developmental epigenetic reprogramming patterns (14). In mouse early preimplantation embryos, a state of low DNA methylation is actively and passively maintained through mechanisms of DNA demethylation, Dnmt1 exclusion, cell division, and Prdm14 expression, while chromatin is dynamic and more dispersed (60,61,63). The organisation of condensed chromatin domains and chromocenters appears after implantation and also in epiblast stem cells, for which DNA methylation is required (63).

We propose that global hypomethylation is a consequence of transcriptional and signalling changes in 2i medium, but is not essential for adaptation of mESCs to the ‘transcriptional ground state’ as defined by core transcription changes that occur irrespective of methylation in the conversion of mESCs to the ground state. This implies that signalling induced gene regulatory networks, and not epigenetic mechanisms, dominate to regulate transcriptional changes required for mESCs to achieve ground state. This is compatible with studies showing that inactivation of many epigenetic regulators (*Ezh2*, *Eed*, *Dnmt1*, *Uhrf1*, *Ring1b*, *G9a*, *Glp*, (*Suv39h1* and *Suv39h2*), *Jarid1b* and *Tet1–3*) is compatible with development up to blastocyst stages and derivation of self-renewing mESCs but incompatible with later stages of development when pluripotency networks are attenuated and alternative differentiation associated GRNs are prominent (64). It is clear that altered patterns of DNA methylation and polycomb in the naive state do not define its identity through gene regulation but may reflect genomic reprogramming to earlier patterns present in pre-blastocyst embryos, with 2i conditions reversing chromatin transitions that may develop into the epigenetic control of gene expression in differentiated cells. A key question is, when do these repressive epigenetic pathways become operational and possibly outweigh signalling cues, and what roles do these early transitions play in subsequent differentiation to stable somatic lineage identities. It is plausible that this is linked with core transcription factor changes enabling essential regulatory roles for epigenetic processes in coordinating and maintaining differential patterns of gene expression.

## DATA AVAILABILITY

Data generated or used in this study can be found NCBI GEO series accession numbers:

- J1 serum/2i MeDIP-seq & 3B3l serum/2i MeDIP-seq: GSM1865089, GSM1865091, GSM2700270, GSM2700271, GSM1865090 & GSM1865092
- WT serum/2i H3K27me3 ChIP-seq GSE23943
- 3B3l serum/2i H3K27me3 ChIP-seq: GSM2700276, GSM2700277
- TKO serum/2i H3K27me3 ChIP-seq: GSM2700278, GSM2700279, GSM2700284 & GSM2700285
- J1, TKO and 3B3l serum/2i gene expression data (microarray): GSE72302
- J1 and 3B3l serum/2i gene expression (RNA-seq): GSE121171
- TKO gene expression (RNA-seq): GSE130686
- EPZ gene expression: GSE101928.

## Supplementary Material

gkaa529_Supplemental_Files

## Notes

Present address: Tuempong Wongtawan, Akkhraratchakumari Veterinary College, Walailak University, 222 Thaiburi, Thasala, Nakhon Si Thammarat 80160, Thailand.
