# Supplementary material for: Activation of transcription factor circuity in 2i-induced ground state pluripotency is independent of repressive global epigenetic landscapes
Source: Nucleic Acids Res. 2020 Jun 25;48(14):7748–66. doi: 10.1093/nar/gkaa529 (PMC7641322; doi:10.1093/nar/gkaa529)
Supplement: gkaa529_Supplemental_Files [file gkaa529_supplemental_files.zip › Shukla et al. Supp Figures.pdf]

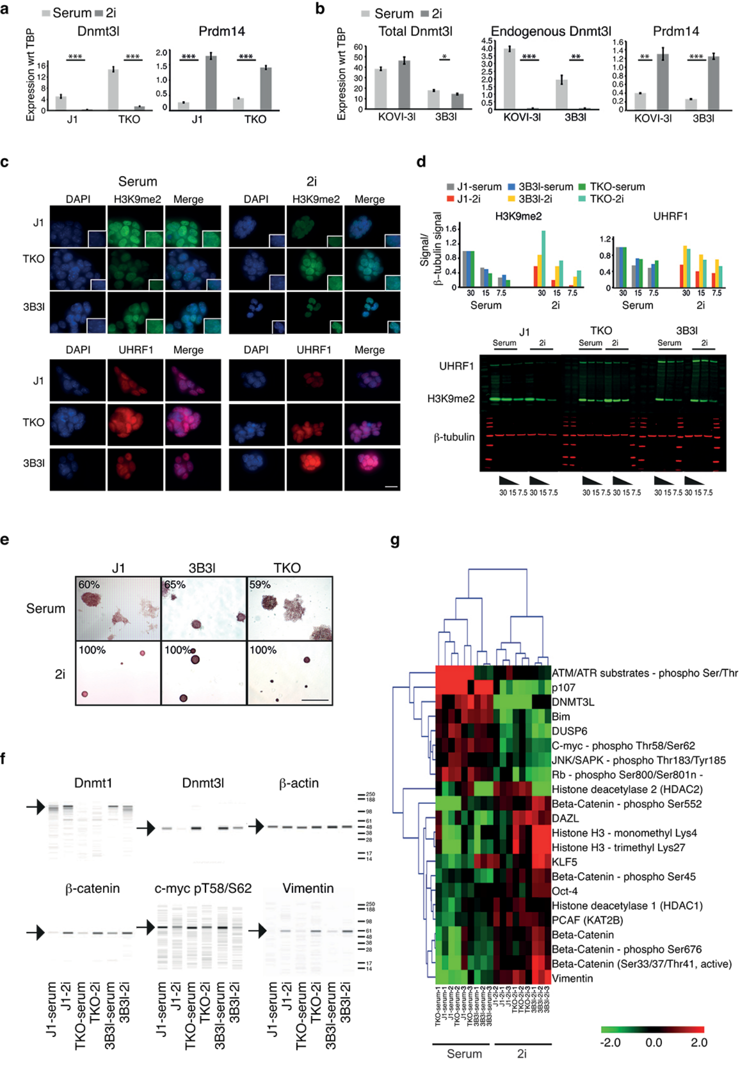
a
Serum 2i
Dnmt3l
Prdm14
Expression wrt TBP
J1
TKO
b
Total Dnmt3l
Endogenous Dnmt3l
KOVI-3l
3B3l
c
Serum
2i
DAPI
H3K9me2
Merge
UHRF1
J1
TKO
3B3l
d
J1-serum
3B3l-serum
TKO-serum
J1-2i
3B3l-2i
TKO-2i
Signal/ β-tubulin signal
H3K9me2
UHRF1
30 15 7.5
UHRF1
H3K9me2
β-tubulin
e
J1
3B3l
TKO
Serum
2i
60%
65%
59%
100%
100%
100%
f
Dnmt1
Dnmt3l
β-actin
β-catenin
c-myc pT58/S62
Vimentin
J1-serum
J1-2i
TKO-serum
TKO-2i
3B3l-serum
3B3l-2i
g
ATM/ATR substrates - phospho Ser/Thr
p107
DNMT3L
Bim
DUSP6
C-myc - phospho Thr58/Ser62
JNK/SAPK - phospho Thr183/Tyr185
Rb - phospho Ser800/Ser801n -
Histone deacetylase 2 (HDAC2)
Beta-Catenin - phospho Ser552
DAZL
Histone H3 - monomethyl Lys4
Histone H3 - trimethyl Lys27
KLF5
Beta-Catenin - phospho Ser45
Oct-4
Histone deacetylase 1 (HDAC1)
PCAF (KAT2B)
Beta-Catenin
Beta-Catenin - phospho Ser676
Beta-Catenin (Ser33/37/Thr41, active)
Vimentin
-2.0
0.0
2.0
Serum
2i

Figure S1: Cell line derivation and characterisation of J1, TKO and 3B3l mESCs in response to 2i culturing

a, Expression analysis of indicated genes by RT-qPCR in TKO and J1 mESCs cultured in serum (light grey) and 2i (dark grey) for 2 weeks. Values represent mean ± S.E. of gene expression wrt (with respect to) the housekeeping gene TBP (TATA Binding Protein) * $p < 0.001$, (unpaired t-test). **b,** Expression analysis of indicated genes by RT-qPCR in KOVI-3l and 3B3l mESCs cultured in serum (light grey) and 2i (dark grey) for 2 weeks. Values represent mean ± S.E. of gene expression wrt the housekeeping gene TBP. * $p < 0.05$,** $p < 0.005$, (unpaired t-test). **c**, Immunocytochemistry of indicated cell lines showing (top) DAPI (blue), H3K9me2 (green) and merge or (bottom) DAPI (blue), UHRF1 (red) and merge. Scale bar is 20μM. **d**, Fluorescent western blots showing H3K9me2 and UHRF1 levels relative to β-tubulin and subtracted for local mean background. Barcharts show a representative quantification of a dilution series of protein 30, 15 or 7.5 μg with corresponding western blots shown below. **e**, Representative images of alkaline phosphatase (AP) staining for indicated cell lines. Numbers indicate the percentage of colonies homogeneously stained for AP in the given condition. **f,** Representative DigiWest blots showing levels of indicated proteins for indicated mESC lines and culture conditions. **g,** Hierarchical clustering (Euclidean distance) and heatmap representing analytes deemed significant (P value < 0.005) after two-factor ANOVA was performed on DigiWest results.

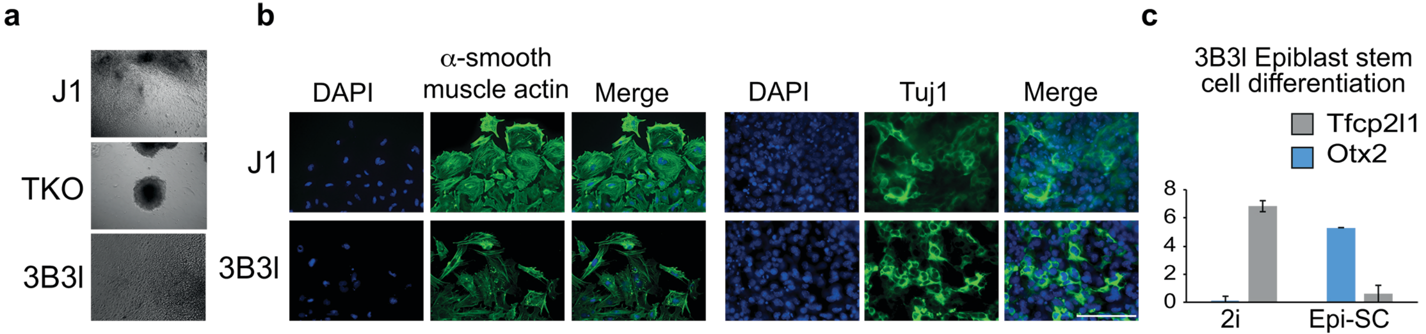

Figure S2: Embryoid bodies generated from J1, TKO and 3B3l mESCs

a, Images of embryoid body outgrowths of indicated cell lines. **b,** Images showing DAPI (blue), a-smooth muscle actin and Tuj1 (green) and merged images of indicated cell lines, scale bar 100 µm. **c**, Expression analysis of indicated genes by RT-qPCR for 3B3l-2i or 3B3l Epiblast-stem cells, values represent mean ± S.E. of expression wrt TBP from 3 technical replicates.

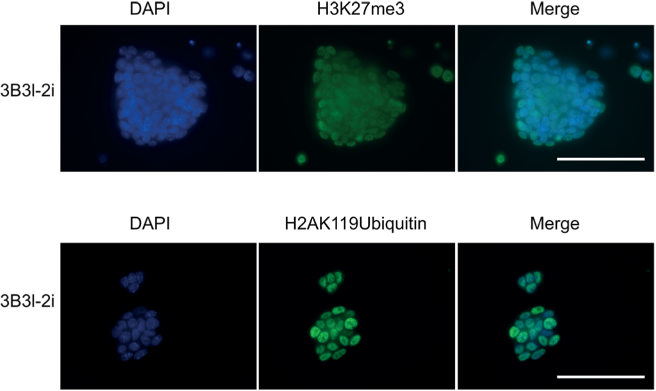

Figure S3: Epigenetic state of heterochromatin in 3B3l mESCs in 2i.

ICC of H3K27me3 (green) and H2AK119Ubiquitin (green) in indicated cells with DAPI (blue) and merge (turquoise represents overlapping signal), scale bar 100µm.

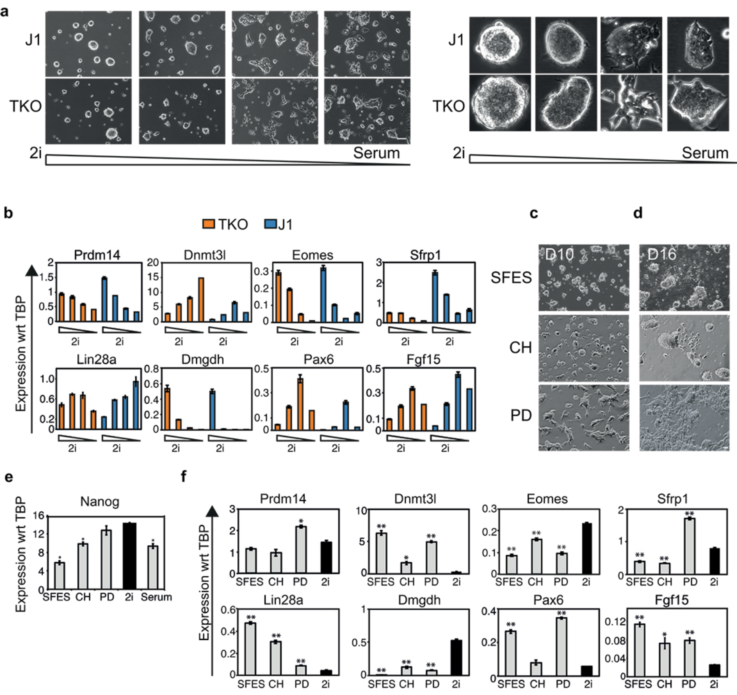

Figure S4: 2i titration and effect of individual inhibitors on J1 and TKO mESCs

a, Morphology images of J1 and TKO cells cultured in either 2i, 1⁄2 2i, 1⁄4 2i or Serum. The panel on the right represents 'zoomed-in' images of a representative colony for each condition. Scale bar 100μm. **b**, Expression analysis of indicated genes by RT-qPCR in TKO (orange) and J1 (blue) mESCs in 2i, 2i (1:2), 2i (1:4) and serum for 2 weeks. Values represent mean ± S.E. of gene expression wrt the housekeeping gene TBP (TATA Binding Protein). **c-d,** TKO cells were cultured in SFES (2i basal media + LIF) with no inhibitors or with 2i or 1i (CH or PD) as indicated. Morphology images of indicated cells after 10 and 16 days in culture, scale bar 100μm. **e**, Expression analysis of Nanog by RT-qPCR after 10 days in culture, values represent mean ± S.E. * $p \leq 0.002$ compared to TKO-2i (unpaired t-test. **f**, Expression analysis of indicated genes by RT-qPCR, values represent mean ± S.E.* $p \leq 0.02$, ** $p \leq 0.0005$ (unpaired t-test) compared to TKO-2i. Experiment was done as biological and technical triplicates; representative dataset shown.

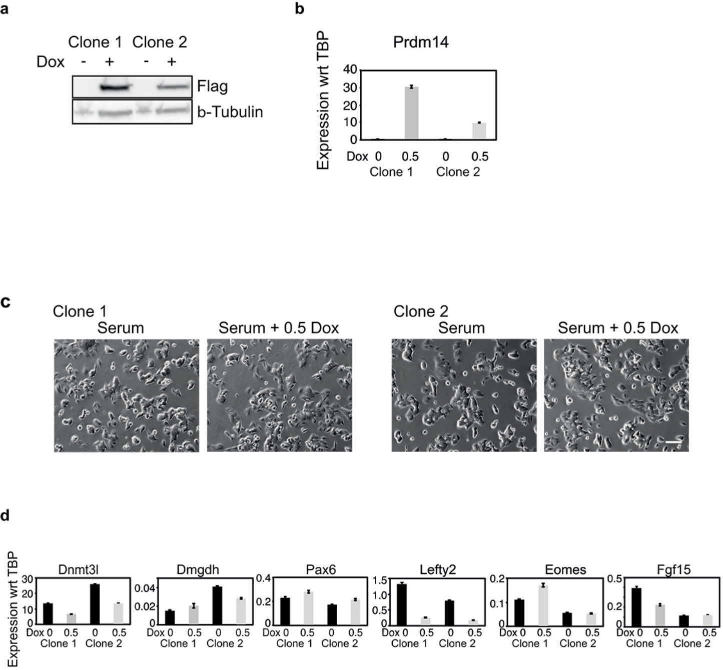

Figure S5: Over-expression of Prdm14 in TKO mESCs

a, Western blot analysis of whole cell protein extract from 2 independent clones of TET-ON-Flag-Prdm14-TKO cells following doxycycline (0.05 mg/ml) induction for 14 days. Blots were probed with M2-anti-Flag antibody to check for induced Prdm14, β-tubulin was used as loading control. **b**, RT-qPCR of Prdm14 to confirm overexpression, values represent mean ± S.E. of expression wrt TBP from 3 technical replicates * $p < 0.0001$ (unpaired t-test). **c**, Morphology images of TET-ON-Flag-Prdm14-TKO cells +/- doxycycline treatment, scale bar 100µm. **d**, RT-qPCR for indicated genes Prdm14-TKO mESCs. Values represent mean ± S.E. of expression wrt TBP of 3 technical replicates. * $p < 0.02$, ** $p<0.002$ (unpaired t-test).

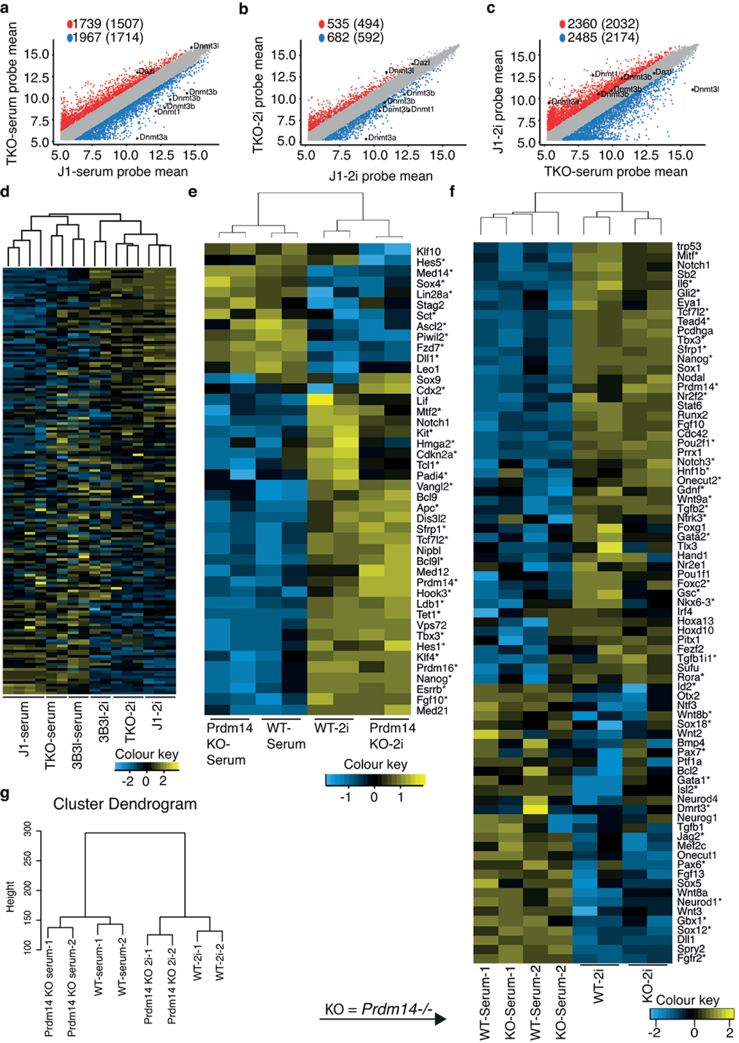
a
1739 (1507)
1967 (1714)
TKO-serum probe mean
J1-serum probe mean
Dnmt3l
Dazl
Dnmt3b
Dnmt3b
Dnmt3b
Dnmt1
Dnmt3a
b
535 (494)
682 (592)
TKO-2i probe mean
J1-2i probe mean
Dazl
Dnmt3l
Dnmt3b
Dnmt3b
Dnmt3b
Dnmt1
Dnmt3a
c
2360 (2032)
2485 (2174)
J1-2i probe mean
TKO-serum probe mean
Dnmt1
Dnmt3b
Dazl
Dnmt3b
Dnmt3b
Dnmt3a
Dnmt3l
5.0
7.5
10.0
12.5
15.0
d
J1-serum
TKO-serum
3B3l-serum
3B3l-2i
TKO-2i
J1-2i
Colour key
-2 0 2
e
Klf10
Hes5*
Med14*
Sox4*
Lin28a*
Stag2
Sct*
Ascl2*
Piwil2*
Fzd7*
Dll1*
Leo1
Sox9
Cdx2*
Lif
Mtf2*
Notch1
Kit*
Hmga2*
Cdkn2a*
Tcl1*
Padi4*
Vangl2*
Bcl9
Apc*
Dis3l2
Sfrp1*
Tcf7l2*
Nipbl
Bcl9l*
Med12
Prdm14*
Hook3*
Ldb1*
Tet1*
Vps72
Tbx3*
Hes1*
Klf4*
Prdm16*
Nanog*
Esrrb*
Fgf10*
Med21
Prdm14 KO-Serum
WT-Serum
WT-2i
Prdm14 KO-2i
Colour key
-1 0 1
f
trp53
Mitf*
Notch1
Sb2
Il6*
Gli2*
Eya1
Tcf7l2*
Tead4*
Pcdhga
Tbx3*
Sfrp1*
Nanog*
Sox1
Nodal
Prdm14*
Nr2f2*
Stat6
Runx2
Fgf10
Cdc42
Pou2f1*
Prrx1
Notch3*
Hnf1b*
Onecut2*
Gdnf*
Wnt9a*
Tgfb2*
Ntrk3*
Foxg1
Gata2*
Tlx3
Hand1
Nr2e1
Pou1f1
Foxc2*
Gsc*
Nkx6-3*
Irf4
Hoxa13
Hoxd10
Pitx1
Fezf2
Tgfb1i1*
Sufu
Rora*
Id2*
Otx2
Ntf3
Wnt8b*
Sox18*
Wnt2
Bmp4
Pax7*
Ptf1a
Bcl2
Gata1*
Isl2*
Neurod4
Dmrt3*
Neurog1
Tgfb1
Jag2*
Mef2c
Onecut1
Pax6*
Fgf13
Sox5
Wnt8a
Neurod1*
Wnt3
Gbx1*
Sox12*
Dll1
Spry2
Fgfr2*
WT-Serum-1
KO-Serum-1
WT-Serum-2
KO-Serum-2
WT-2i
KO-2i
Colour key
-2 0 2
g
Cluster Dendrogram
Height
100
150
200
250
300
Prdm14 KO serum-1
Prdm14 KO serum-2
WT-serum-1
WT-serum-2
Prdm14 KO 2i-1
Prdm14 KO 2i-2
WT-2i-1
WT-2i-2
KO = Prdm14-/-

Figure S6: Expression array analysis of J1, TKO, WT and Prdm14$^{-/-}$ (KO) cells in serum and 2i. Prdm14 WT and KO Data is a subset of GEO Series GSE42580.

a-**c**, Scatter plot of probes (genes) shown as log2 expression values, red and blue dots indicate differ - entially expressed probes (FC ≥ 2, p ≤ 0.05) and grey dots = unchanged expression. Selected genes have been highlighted to illustrate the difference between the two cell types. Numbers on top represent differentially expressed probes; numbers in parenthesis represent the equivalent number of genes a, TKO-serum versus J1-serum b, TKO-2i versus J1-2i c, J1-2i versus TKO-serum. **d,** Heatmap for all the probes related to the GO term ‘Stem Cell Maintenance’ in indicated cell types. **e**, Heatmap repre - senting expression levels of stem cell maintenance genes differentially expressed (FC ≥ 1.5) in either WT or Prdm14$^{-/-}$ mESCs in 2i compared to their serum counterparts. **f**, Heatmap representing expres - sion levels of cell fate commitment genes differentially expressed (FC ≥ 2) in WT cells in 2i compared to serum conditions. * Indicates genes overlapping with datasets of present study. **g**, Hierarchical clustering of the expression array data including all the probes (Euclidean distance and Ward.D2 method).

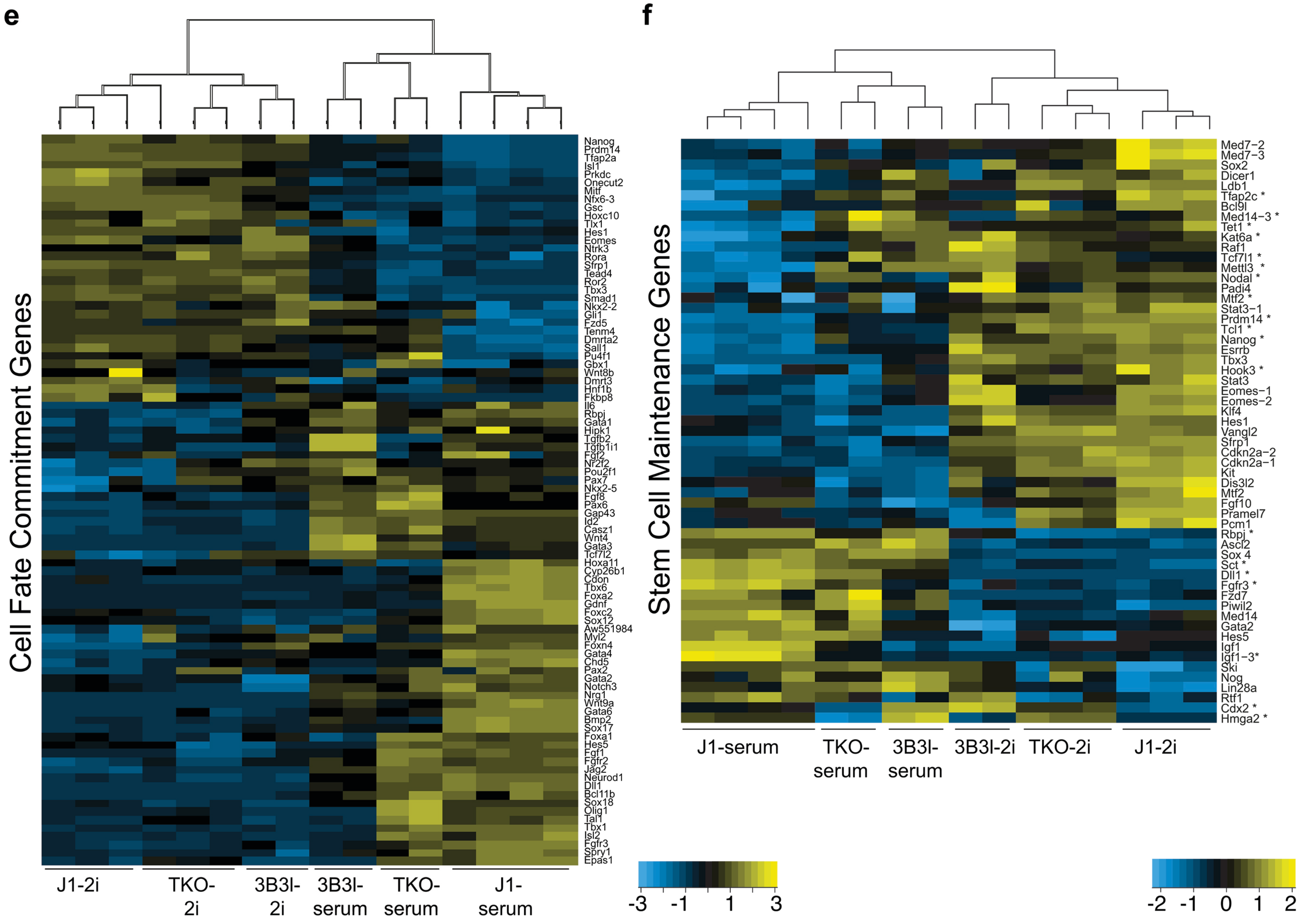

Figure S7 derived from Fig. 3. Gene expression changes in mESCs upon culturing in 2i e-d.
A portion of Figure 3 was enlarged so that the gene names on the right can be read.
e, Heatmap representing expression levels of 'Cell Fate Commitment' genes differentially expressed (FC ≥ 1.5, p.adj. ≤ 0.05 eBayes (limma), Benjamini-Hochberg corrected) in J1-2i compared to J1-serum in indicated cells. * marks genes which are differentially expressed (FC ≥ 2, p.adj. ≤ 0.05 eBayes (limma), Benjamini-Hochberg corrected) between TKO-serum and J1-serum. **f**, Same as e for 'Stem Cell Maintenance' genes.

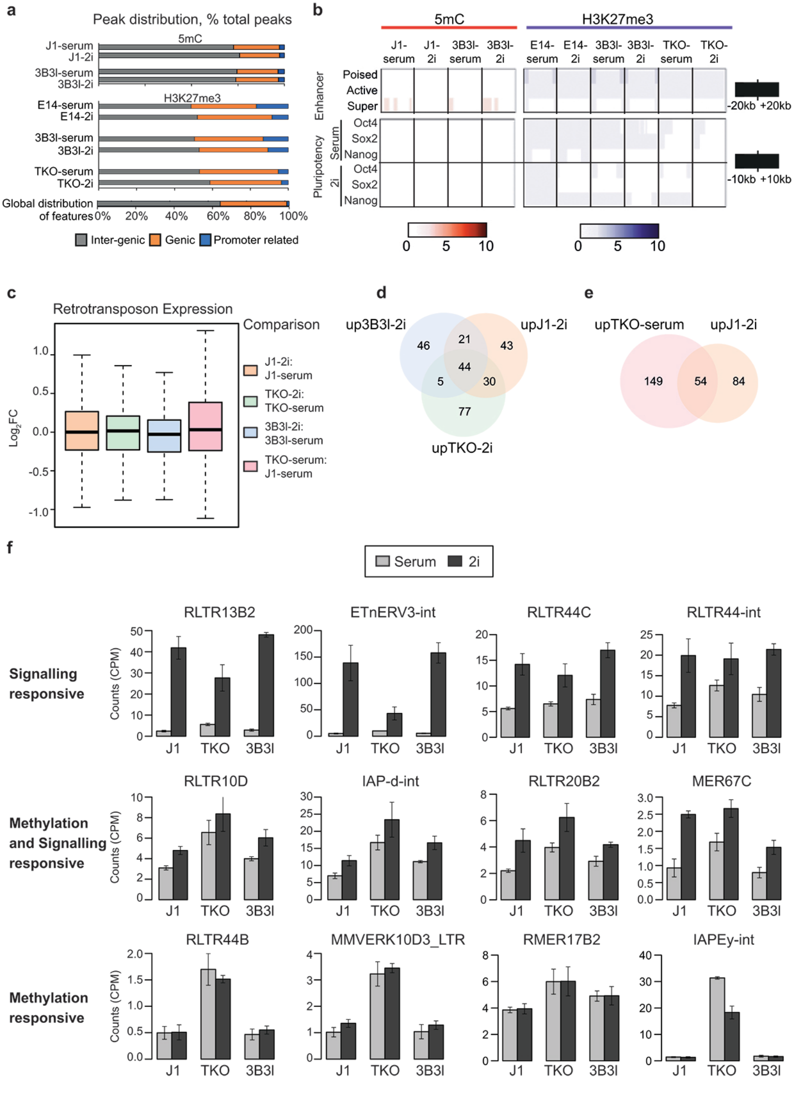

a
Peak distribution, % total peaks
5mC
J1-serum
J1-2i
3B3l-serum
3B3l-2i
H3K27me3
E14-serum
E14-2i
3B3l-serum
3B3l-2i
TKO-serum
TKO-2i
Global distribution of features
0% 20% 40% 60% 80% 100%
Inter-genic Genic Promoter related
b
5mC
H3K27me3
J1-serum J1-2i 3B3l-serum 3B3l-2i
E14-serum E14-2i 3B3l-serum 3B3l-2i TKO-serum TKO-2i
Enhancer
Poised
Active
Super
Pluripotency
Serum
2i
Oct4
Sox2
Nanog
-20kb +20kb
-10kb +10kb
0 5 10
c
Retrotransposon Expression
Comparison
J1-2i: J1-serum
TKO-2i: TKO-serum
3B3l-2i: 3B3l-serum
TKO-serum: J1-serum
Log2FC
1.0 0.5 0.0 -0.5 -1.0
d
up3B3l-2i
upJ1-2i
upTKO-2i
46 21 43 44 5 30 77
e
upTKO-serum
upJ1-2i
149 54 84
f
Serum 2i
Signalling responsive
Methylation and Signalling responsive
Methylation responsive
Counts (CPM)
RLTR13B2
ETnERV3-int
RLTR44C
RLTR44-int
RLTR10D
IAP-d-int
RLTR20B2
MER67C
RLTR44B
MMVERK10D3_LTR
RMER17B2
IAPEy-int
J1 TKO 3B3l

Figure S8: Genome wide analysis of 5mC and H3K27me3 peak distribution in mESCs in serum and 2i.

a, Plot of the distribution of 5mC and H3K27me3 peaks for indicated cell lines and conditions, promoters (TSS +/- 1kb: blue), genic (orange), inter-genic (grey). **b**, Heatmap indicating 5mC (red) and H3K27me3 (purple), levels; 0-10, see scale, for indicated cell lines and conditions across pluripotency transcription factor binding elements, Oct4, Sox2 and Nanog, +/- 10kb. **c**, Boxplot showing changes in the abundance (Log2FC: log2 fold change) of retrotransposon RNAs (LINE, SINE and LTR Repeatmasker classes) in the indicated comparisons between cell lines and conditions. **d-e**, Overlaps between sets up retrotransposons upregulated (logFC > 0 and FDR < 0.05) in the indicated comparisons. **f**, Behaviour of selected retrotransposon RNAs between the indicated cell lines and conditions. Mean RNA abundance (CPM, counts per million mapped reads) and standard deviation between three replicates for each sample are indicated. Signalling responsive retrotransposons are upregulated in 2i in J1, TKO and 3B3l cells, i.e. regardless of the methylation state of the cell line. Methylation responsive retrotransposons are upregulated in hypomethylated (TKO) mESCs in serum relative to J1 mESCs in serum, but not in 3B3l cells in response to 2i. Retrotransposons responding to 2i conditions and to the methylation state of the cell are upregulated in 2i in J1, TKO and 3B3l cells, but the level of retrotransposon expression is affected by the methylation state of the cell (i.e. upregulated in TKO cells relative to 3B3l cells). Some of the retrotransposons identified in this analysis (RLTR13B2::EtnERV3-int, RLTR44C::RLTR44-int, RLTR10D::IAP-d-int) correspond to long terminal repeats and their cognate adjacent internal sequences that they can be found flanking in the mouse genome.

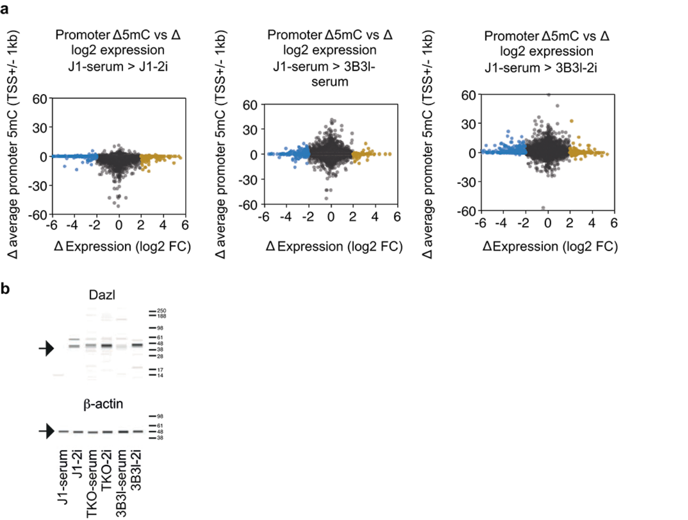

Figure S9: Analysis of relationship between gene expression changes and locus specific 5mC levels.

a, Scatter plots of change in average promoter 5mC levels against change in gene expression (log2 fold change) for indicated cell lines and culture conditions. Blue = downregulated genes, yellow = upregulated, grey = no change. **b**, Representative DigiWest Blot for Dazl and β-actin in indicated cell lines and culture conditions.

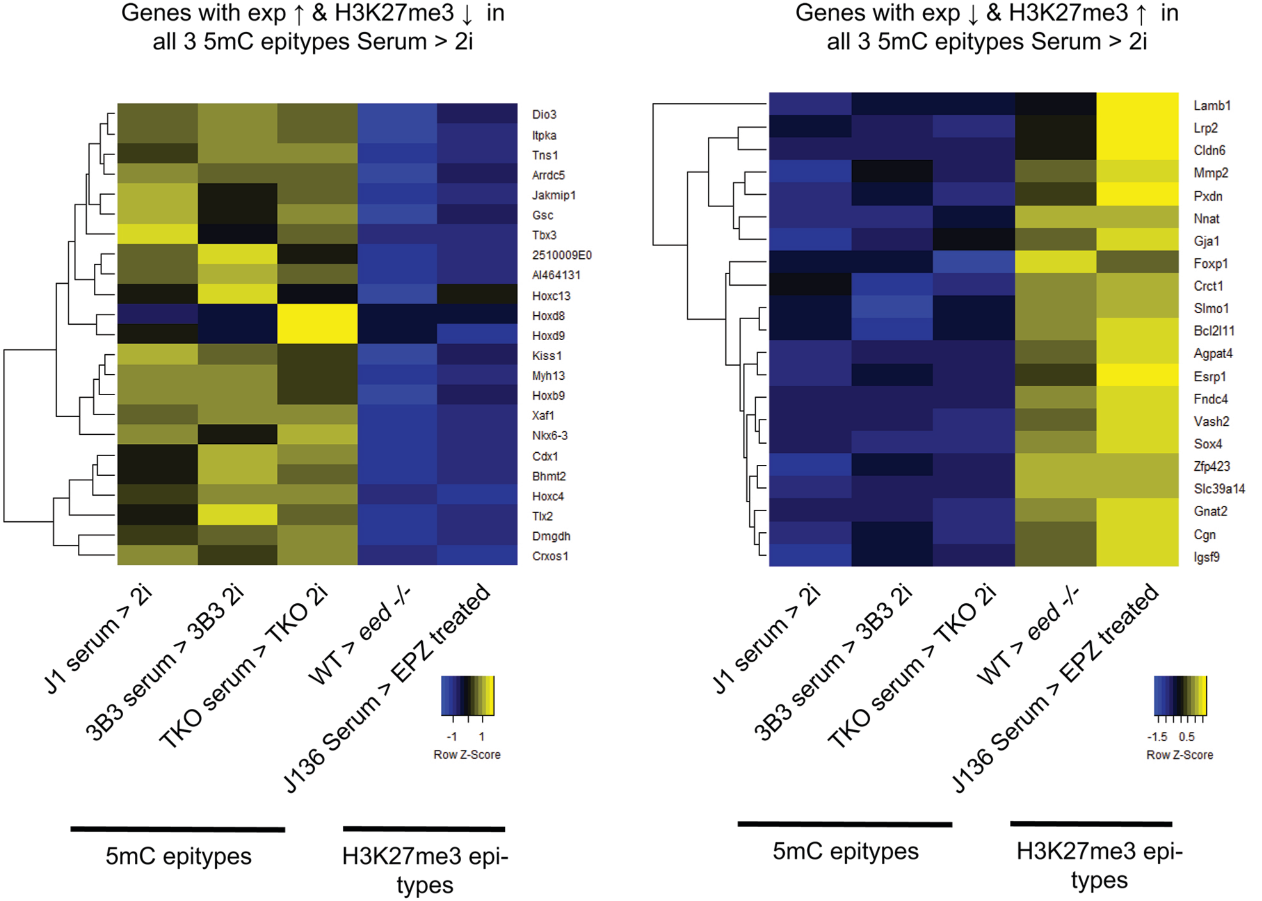

Figure S10: Z-score normalised heatmap of gene expression changes between serum to 2i of genes that display opposite expression and H3K27me3 changes: conserved across all three 5mC epitypes (J1, TKO & 3B3l – all serum to 2i) and their response to PRC2 inactivation (*eed*$^{-/-}$ and EPZ6438 inhibition (of EZH2 activity) in serum.

By comparison cell types in which the polycomb machinery are perturbed (WT mESC in serum vs *eed*$^{-/-}$ cells in serum) or inhibited (J136 mESC in serum vs J136 mESC treated with EPZ6438) do not show similar gene expression changes. Plots show data over serum to 2i gene expression gains/H3K27me3 losses (top) or over serum to 2i gene expression losses/H3K27me3 gains (bottom). Yellow: relative elevation in gene expression, blue: relative reduction in gene expression.

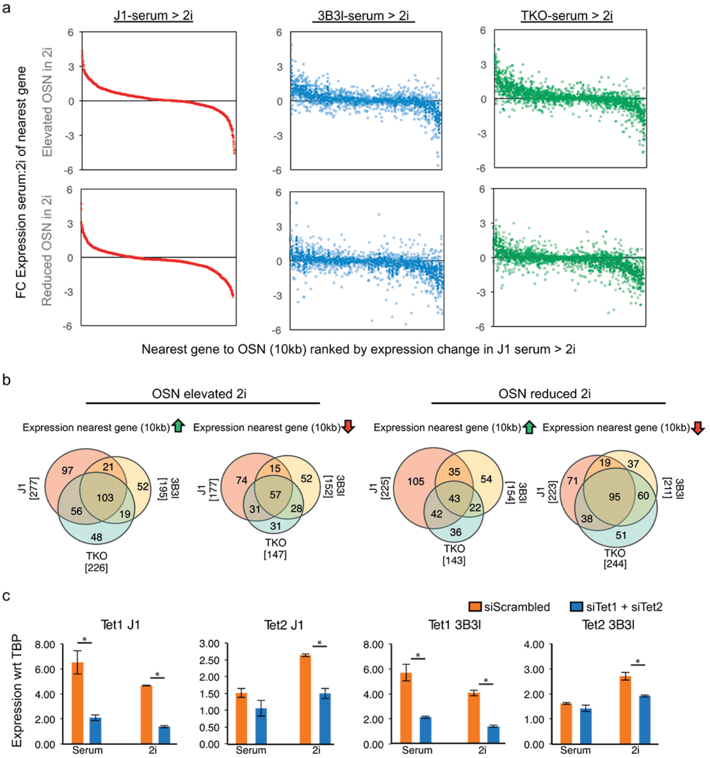

Figure S11: Relationship between, transcript levels, epigenetic marks and culture conditions in mESCs.

a, Scatterplots showing fold change gene expression 2i versus serum of the nearest gene within 10kb of an OSN site that has elevated OSN factor binding in 2i versus serum (top row), or reduced OSN factor binding in 2i versus serum (bottom row) for indicated cell lines. Data has been ranked by expression change in J1 serum versus 2i. Patterns of expression change (2i-serum) are similar at genes nearest an OSN gained or lost upon 2i transition; in all three cell lines. Serum and 2i specific OSN sites are taken from published datasets (see methods). **b**, Venn diagrams showing the overlap for indicated cell lines of the same gene set as in panel A. The left panel shows OSN sites that have increased binding of OSN factors in 2i, where this corresponds to an increase in gene expression of the nearest gene within 10kb (left) and where this is coincident with reduced gene expression (right). The right panel shows OSN sites that have reduced binding of OSN factors in 2i relative to serum, where gene expression of the nearest gene within 10kb either increases (left) or is reduced (right). **c,** Expression analysis of indicated genes by RT-qPCR in J1 and 3B3l mESCs in 72h serum or 48h serum + 24h 2i transfected with either scrambled siRNA (orange) or siRNAs for Tet1 + Tet2 (blue). Values represent mean +/- S.E. of 2 biological replicates, which consist of three technical replicates each with respect to (wrt) to the housekeeping gene TBP (TATA Binding Protein). *$p < 0.05$ (unpaired t-test).
