# Supplementary material for: Activation of transcription factor circuity in 2i-induced ground state pluripotency is independent of repressive global epigenetic landscapes
Source: Nucleic Acids Res. 2020 Jun 25;48(14):7748–66. doi: 10.1093/nar/gkaa529 (PMC7641322; doi:10.1093/nar/gkaa529)
Supplement: gkaa529_Supplemental_Files [file gkaa529_supplemental_files.zip › Shukla_Supplementary Information Contents page.docx]

Supplementary information includes:

- 11 Supplementary figures and legends
- 3 Supplementary tables:

Table S1: List of primer and siRNA sequences

Table S2: List of antibodies

Table S3: MIQE guidelines

- 2 Datasets:

Dataset 1: Affymetrix expression array data and GO term analysis

Dataset 2: Luminex reads, Digiwest protein analysis

- 1 word document with supplementary table legends
