# Supplementary material for: Activation of transcription factor circuity in 2i-induced ground state pluripotency is independent of repressive global epigenetic landscapes
Source: Nucleic Acids Res. 2020 Jun 25;48(14):7748–66. doi: 10.1093/nar/gkaa529 (PMC7641322; doi:10.1093/nar/gkaa529)
Supplement: gkaa529_Supplemental_Files [file gkaa529_supplemental_files.zip › Shukla_Supplementary_Table_and_Dataset_Legends_2020.docx]

**Supplementary Table and Dataset Legends**

**Table S1:** List of primers utilised for RT-qPCR and bisulfite-PCR. All RT-qPCR primers span at least one intron except for FlagDnmt3l. Primer specificity was determined by NCBI Primer-Blast software.

**Table S2:** List of antibodies used for immunocytochemistry, western blotting, ChIP and DigiWest analysis.

**Table S3:** Details of MIOQE guidelines

**Dataset 1:** **Affymetrix expression array data of the cells profiled in this study.**

Table includes Log_2_ expression values for each probe in indicated cell types. Also lists of differentially expressed genes (FC ≥ 2, p < 0.05) are indicated along with GO term enrichment analysis using Panther gene analysis tools (pantherdb.org). Also probes related to the GO term: ‘Stem Cell Maintenance’ (SCM) and the GO-term: ‘Cell Fate Commitment’ (CFC) are listed.

See Excel file.

**Dataset 2:** **DigiWest data from cells profiled in this study.** DigiWest measurements were performed on total cell lysates. Peaks detected at the expected molecular weight were integrated and the determined values are listed for all used antibodies. See Excel file.
